# Developmental and transcriptional responses of maize to drought stress under field conditions

**DOI:** 10.1002/pld3.129

**Published:** 2019-05-07

**Authors:** Olga N. Danilevskaya, GongXin Yu, Xin Meng, John Xu, Elizabeth Stephenson, Stacey Estrada, Sunita Chilakamarri, Gina Zastrow‐Hayes, Shawn Thatcher

**Affiliations:** ^1^ DuPont Pioneer Johnston Iowa; ^2^ Iowa Institute of Human Genetics University of Iowa Iowa City Iowa; ^3^ Syngenta Crop US Slater Iowa; ^4^ Indigo Agriculture Charlestown Massachusetts; ^5^ USDA ARS Plant Introduction Research Unit Ames Iowa

**Keywords:** abiotic stress, development, drought, maize, RNA‐seq

## Abstract

Drought is a common abiotic stress which significantly limits global crop productivity. Maize is an important staple crop and its yield is determined by successful development of the female inflorescence, the ear. We investigated drought stress responses across several developmental stages of the maize B73 inbred line under field conditions. Drought suppressed plant growth, but had little impact on progression through developmental stages. While ear growth was suppressed by drought, the process of spikelet initiation was not significantly affected. Tassel growth was reduced to a lesser extent compared to the observed reduction in ear growth under stress. Parallel RNA‐seq profiling of leaves, ears, and tassels at several developmental stages revealed tissue‐specific differences in response to drought stress. High temperature fluctuation was an additional environmental factor that also likely influenced gene expression patterns in the field. Drought induced significant transcriptional changes in leaves and ears but only minor changes in the tassel. Additionally, more genes were drought responsive in ears compared to leaves over the course of drought treatment. Genes that control DNA replication, cell cycle, and cell division were significantly down‐regulated in stressed ears, which was consistent with inhibition of ear growth under drought. Inflorescence meristem genes were affected by drought to a lesser degree which was consistent with the minimal impact of drought on spikelet initiation. In contrast, genes that are involved in floret and ovule development were sensitive to stress, which is consistent with the detrimental effect of drought on gynoecium development and kernel set.

## 1 INTRODUCTION

Drought is the most common abiotic stress limiting global crop productivity (Boyer et al., 2013). Exceptionally severe drought conditions occur in approximately 25‐year cycles, but almost every crop growing season has periods of mild to moderate dry conditions (Mallya, Zhao, Song, Niyogi, & Govindaraju, 2013). The 2012 North American drought significantly reduced yield of all crops, with maize experiencing the greatest yield loss (Mallya et al., 2013). Developing maize hybrids with enhanced drought tolerance either through conventional breeding (Cooper, Gho, Leafgren, Tang, & Messina, 2014) or biotechnology (Deikman, Petracek, & Heard, 2012; Habben et al., 2014; Mittler & Blumwald, 2010; Shi et al., 2015) is a goal of many maize improvement programs. Understanding the underlying physiological and genetic processes controlling maize reproductive development under water‐limited conditions is crucial to this goal.

Among the major cereal crops, maize is the only monoecious plant bearing unisexual flowers. The male inflorescence, or tassel, develops from the shoot apical meristem at the top of the plant, whereas the female inflorescence, or ear, develops from lateral meristems in the axil of leaves. The initial steps of development in both inflorescences are very similar; however, sex related differences appear at later developmental stages (Cheng, Greyson, & Walden, 1983). The basic unit of the ear inflorescence is the pistillate spikelet, composed of a floret subtended by a pair of glumes. Development of the ear is defined by several meristem transitions starting with spikelet pair meristems (SPMs) arising from the flanks of each inflorescence meristem (IM). Each SPM gives rise to two spikelet meristems (SMs) which terminate as a floral meristem (FM) (Cheng et al., 1983; Tanaka, Pautler, Jackson, & Hirano, 2013). The FM produces different organ primordia, including the gynoecium (female reproductive structure) which terminates in formation of an ovary composed of the embryo sac and the silk, a stigmatic structure. The ear is protected by several layers of husks, which are modified leaves. When silks exert from the husk, the female flowers are fertilized by pollen shed from the tassel and kernel development commences.

In the US Corn Belt the most severe consequences of drought occur at flowering time (Shaw, 1977) and result in multiple detrimental effects on the female inflorescence and to a lesser extent negative effects on the male inflorescence (Herrero & Johnson, 1980a). Silk elongation is dramatically reduced under limited water conditions due to a reduction in cell division and cell expansion (Fuad‐Hassan, Tardieu, & Turc, 2008). The delay of silk extrusion results in a longer ASI (anthesis to silking interval) and can reduce pollination efficiency (Araus, Serret, & Edmeades, 2012). Even short periods of water deficits can lead to abnormalities in embryo sac development (Moss & Downey, 1971), as well as zygotic and early kernel abortion (Schussler & Westgate, 1990). For these reasons drought conditions occurring just before and shortly after pollination have the most profoundly negative effect on kernel set and final grain yield (Araus et al., 2012). In contrast, drought episodes during the grain filling period typically have a less severe impact on yield (Barker et al., 2005).

Despite the importance of maize ear growth and development under drought conditions, molecular and genomic data for this organ are surprisingly limited. Several RNA profiling expression studies under drought conditions have been conducted on maize seedlings or leaves (Avramova et al., 2015; Badicean, Scholten, & Jacota, 2011; Fernandes, Morrow, Casati, & Walbot, 2008; Shan et al., 2013; Yue, Zhuang, Li, Sun, & Zhang, 2008; Zheng et al., 2010). There are reports on the drought stress response in maize reproductive tissues including pre‐pollinated ears (Zinselmeier et al., 2002), ears, and silks (Li et al., 2007; Zhuang et al., 2007), as well as ovaries and developing kernels (Kakumanu et al., 2012; Luo, Liu, Lee, Scully, & Guo, 2010; Marino et al., 2009). Most of these studies were conducted in a controlled environment and led to important discoveries of stress response in maize. However controlled environments lack fluctuating meteorological conditions such as solar radiation, heat, wind, and vapor pressure deficit, which play key roles in plant growth and development. The complexity of natural environments emphasizes the importance of conducting drought experiments in the field (Blum, 2014). To address this issue, we designed and conducted two drought stress experiments under field conditions. The first experiment was located at Woodland, California (USA), which has been developed as a managed‐stress environment for phenotyping the drought response of maize (Campos, Cooper, Habben, Edmeades, & Schussler, 2004). Drought stress was imposed over 1 month with the maximum intensity of stress occurring around flowering time. A second experiment was conducted in Johnston, Iowa (USA), within the Corn Belt region. Drought conditions were created by growing maize in pots located in the field and precisely limiting water application. For both experiments, we collected vegetative and reproductive phenology data to understand the tissue specific response to drought stress.

To complement the observations of phenotypic responses, RNA‐seq profiling of leaves, ears, and tassels was conducted to determine relative differences in transcriptomic responses between fully irrigated and drought stressed plants. Overall, these field drought experiments revealed a tissue specific response to stress at both the phenotypic and transcriptional levels, as well as significant flexibility of gene expression in response to changing environmental conditions.

## 2 MATERIALS AND METHODS

### 2.1 Experimental design and drought stress management in the field at Woodland, CA

The B73 inbred line was used for this study because of the public availability of the annotated reference genome (http://www.maizegdb.org/assembly/). The initial drought field experiment was conducted in Woodland, CA (38° 40′N, 121° 50′W) during the summer of 2012. This location typically has no rainfall during the growing season and precision irrigation is provided via sub surface drip tapes. B73 seeds were planted in 4‐row plots in a randomized complete block design with four replications. The length of the plot was 4.4 m, 29 plants per plot. Plant density was 82 K plants per ha. Standard agronomic practices were used, as described in Gaffney et al., 2015. Well‐watered (WW) plots were fully irrigated throughout the entire growth cycle of the crop. In contrast, plots that were designated to receive the drought treatment (DRT) were fully irrigated until plants reached the V7–V8 stage, when irrigation was terminated for the remainder of the experiment. Due to the high water holding capacity of the soil (Yolo silt loam) at this location, plants in this drought stress treatment continued to develop for several weeks after the irrigation was stopped. Daily high and low temperatures were obtained from weatherunderground.com. The nearest weather station (ID: KCAWOODL9) is located 3.6 miles away from the Woodland field.

### 2.2 Tissue sampling for RNA‐seq profiling in the field study

Tissues were sampled from plants of the middle two rows of each plot in both the WW and DRT treatments. The middle portions of the 10th–11th leaves from three plants were cut and these tissues were pooled to make a biological replicate. Leaves were sampled at the peak of stress in the afternoon between 14:00–15:00 p.m. from both well‐watered and drought plots during the diurnal stress period. Within each plot, ears of uniform size were selected and pooled as the biological replicate. Prior to sampling ears, shoot bags were used to cover exposed prophylls prior to silk emergence. For the first sampling at V10–V12 (designated as V12), 32 ears <0.5 cm in length were pooled per plot. For the second sampling at V12–V14 (designated as V14), 16 ears 0.8–1.5 cm in length were pooled per plot. For the third sampling at V16–V18(designated as V18), 3–4 ears that were 3–4 cm in length under WW and 2–3 cm in length under DRT were pooled per plot. For the final sampling at V18–R1(designated as R1), 3–4 ears that were 7–8 cm in length under WW and 3–4 cm in length under DRT were pooled. In the drought stressed plants, the R1 designation based on the timing of silk emergence in well‐watered plants, as the drought stressed ears did not exert silk beyond the husk at this stage. Visible silks were manually removed from ears at the time of sampling and whole ears were used. Tassels were also sampled from the same plants used for ear sampling. The 10 cm middle section of the main tassel spike was removed for the tassel tissue sample. Tassels were still hidden in the whorl at the first (V12) and second (V14) samplings. Tassel emergence occurred at the third (V18) sampling. Shedding was initiated just prior to the fourth (R1) sampling. For all samples, tissues were immediately frozen in liquid nitrogen in the field.

### 2.3 Experimental design and drought stress management in the field pot study

The second field study was conducted in Johnston, IA (41°40′N, 93°42′W) during the summer of 2013. Plants were grown in pots that were arranged in double rows in wooden racks. The plant distance within a row was 20 cm. The row space between the two rows in the rack was 24 cm. The row space between racks was 110 cm. This arrangement produced 26 plants per 4.4 m which is close to the number of plants in a field plot (29 plants/4.4 m). To prevent root growth into soil, pots were supported above the ground on the wooden racks. Drainage holes in pots were covered by paper towels. Pots were covered with plastic bags to prevent rain water entry. The pots were laid out in a split plot (strip‐plot) design with 24 reps per stage equally divided between WW and DRT treatments. Square tree pots (Stuewe&Sons TP818) of 10 L were filled with potting soil (Fafard^®^ 3B mix). All pots for the DRT treatment were filled by weight with the same amount of soil. Water was provided to each pot via irrigation drip tubes and Peters^®^ Excel fertilizer was used with a final N concentration of 100 ppm delivered to the plants. The fertilizer contained 15% N, 5% P_2_O_5_, 15% K_2_O, 5% Ca, 2% Mg, 0.0187% B, 0.0187% Cu, 0.075% Fe, 0.0375% Mn, 0.0075% Mo, and 0.0375% Zn. Plants were grown under full irrigation until they reached the V7–V8 stage. Irrigation was stopped from the DRT pots until 50% of the plants showed obvious leaf wilting. Pots were then re‐watered to full soil capacity. This drying/rehydration cycle was repeated eight times sequentially from stage V8 to R3 over the course of the experiment.

### 2.4 Phenotypic data collection in the field pot study

Growth stages were defined according to the book “Corn growth and development” (Abendroth, Elmore, Boyer, & Marlay, 2011). Plant height was measured from the plant base to the collar of the top fully expanded leaf. Plant height and leaf number were recorded weekly. Pollen shed and silk emergence were recorded on a per plant basis. Ears were dissected from V10 to V14 in the lab and analyzed under a dissecting microscope. At the R1–R3 stages, measurements were done without a microscope. Ear length was measured from the base to the tip. Two rows of spikelets at the opposite sides of the ear were counted at every stage to determine total ovaries/kernels per row.

### 2.5 Growing degree day

Growing degree day was calculated using the standard formula GDD_C_ = (*T*_max_ + *T*_min_)/2) − *T*_base_ where *T*_max_ and *T*_min_ are the daily high and low temperatures (Celsius). The base temperature (*T*_base_) of maize was 10°C (Abendroth et al., 2011; Yang, Logan, & Coffey, 1995).

### 2.6 Statistical analysis

A linear regression formula with *R* ^2^ value (Microsoft Excel) was used to determine the plant growth rate, the leaf appearance rate, the ear elongation rate, and spikelet initiation rate. *T* tests were calculated using the Microsoft Excel 2015 Analysis ToolPak add‐in. All data were analyzed with the *t* test: Two‐Sample Assuming Equal Variances using a two‐tail approach and a *p*‐value of 0.01.

### 2.7 mRNA‐seq library preparation and transcriptome data analysis

Total RNA isolation and deep cDNA sequencing was performed on the Illumina HiSequation 2500 system as described previously (Thatcher et al., 2014, 2016). The generated RPKtM (**R**elative **p**arts per **k**ilobase per **10 m**illion) data matrix was visualized and analyzed in Genedata Analyst^™^ software (Genedata AG, Basel, Switzerland). Genedata Expressionist^®^ for Genomic Profiling was used for RNA‐seq data analysis and visualization. Several statistical applications such as *t* test, ANOVA, linear models, and Principal Components Analysis (PCA) are built in this software. RPKtM values were normalized base‐2 logarithm before all statistical analysis. The total number of expressed genes was detected by mapping reads to maize B73 reference genome sequence V2 as described in (Thatcher et al., 2016). One‐way ANOVA analysis was performed to detect differentially expressed (DE) genes at a Q‐value of 1E‐6 with a combined effect of developmental stages and drought treatment. False discovery rate was corrected for by using a multiple hypothesis testing method (Benjamini, Drai, Elmer, Kafkafi, & Golani, 2001). A default of 1E‐6 FDR was used for selecting of genes that are significantly affected by drought and stage. DE genes with a q‐value of 1E‐6 were designated as significant. PCA, a statistical procedure that models the variation in terms of its principal components, was used to reveal the impact of drought stress and developmental stage on DE genes by tissue. Student's *t* tests were applied to identify genes significantly affected by drought stress at each of the four developmental stages where a false discovery rate of 1E‐2 was used as a cutoff. The stage specific DE genes were subjected to K‐means clustering with positive correlation distance to identify up‐ or down‐regulated genes. Enriched function analysis was performed with GO Fisher's exact test (GOFET, *p*‐value of 0.01) with biological processes as the default main ontology. GOFET is an integrated part of Transcriptome Data Analysis of the software package Genedata Analyst^™^.

### 2.8 Quantitative RT‐PCR

Quantitative RT‐PCR amplifications were performed using TaqMan^®^ probe based detection system (Applied Biosystems). The quantity of target genes was determined by the standard curves of cDNAs pooled from the tissues where the genes are highly expressed. The relative expression level of genes was calculated by their quantification normalized to ubiquitin 5. The processes were performed following the User Bulletin #2 from Applied Biosystems at http://www3.appliedbiosystems.com/cms/groups/mcb_support/documents/generaldocuments/cms_040980.pdf.

### 2.9 Accession numbers

Sequence data from this article can be found in National Center for Biotechnology Information Gene Expression Omnibus under accession number GSE71723.

## 3 RESULTS

### 3.1 Phenology of plants under well‐watered and drought stressed conditions

**Figure 1:**
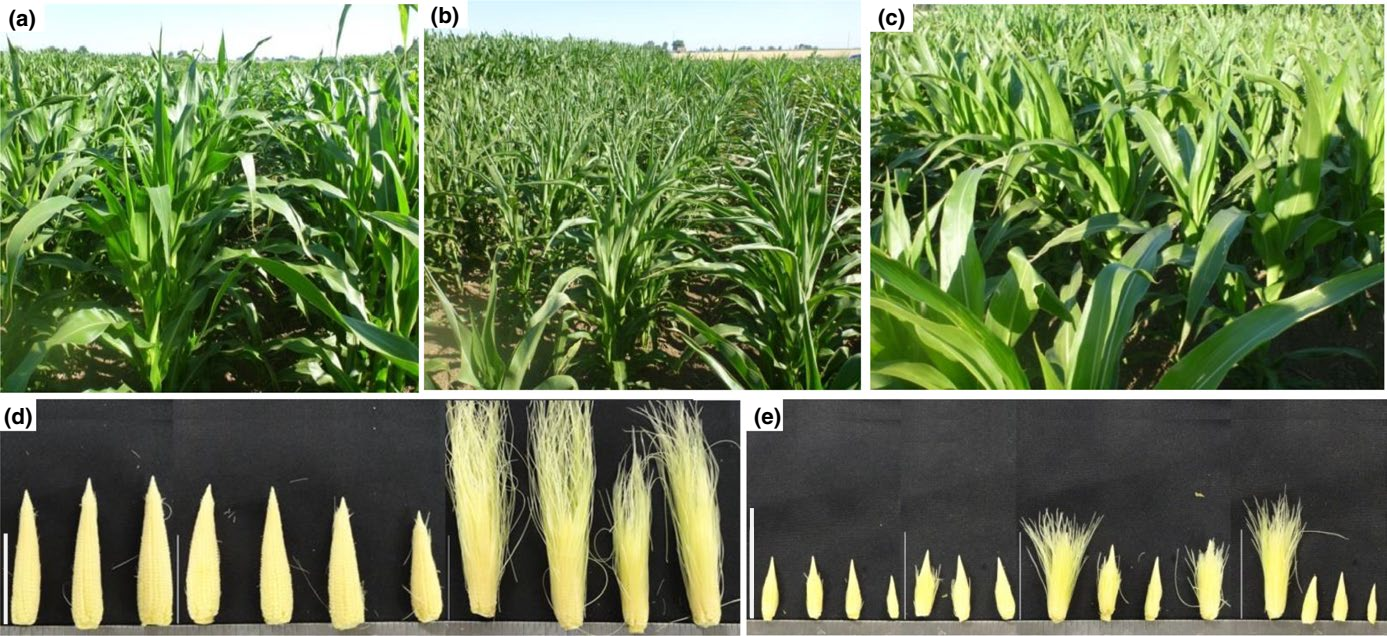
Drought stress experiment in Woodland CA, 2012. WW (a) and DRT (b) plants at 10 days after drought initiation on June 18, 2012 at 18:00 when the high daily temperature was 40°C. DRT plants showed severe leaf wilting. Overnight DRT plants recovered from stress (c) June 19, 2012 at 10:30 when the low nightly temperature was 16°C. Representative ear images (d) without and with silk at the R1 stage (WW) and DRT ear images (e) with intact silk sampled at the same time as WW ears. Scale bars are 5 cm

The initial field experiment was conducted during the summer of 2012 in Woodland, CA in a randomized complete block design. All plots were fully irrigated until plants reached the V7–V8 stage; thereafter, in the drought (DRT) treatment plots irrigation was terminated. The well‐watered (WW) plots were fully irrigated during the remainder of the experiment, and no rainfall occurred during the treatment portion of the study (Supporting Information Figure S1). The first sampling was 11 days after the irrigation was terminated. During these first 11 days, the temperature reached a high of 40°C, imposing heat stress on the plants. At the first sampling (June 19th), no visible signs of stress were observed in the WW plots (Figure 1a). However, a water deficit response was observed in the DRT plots in the form of plant leaf rolling and leaf wilting (Figure 1b) which is a typical manifestation of the low leaf water potential (Fernandez & Castrillo, 1999). The week before the second sampling (June 27th), the temperature was milder, dropping to approximately 26°C. The temperature slowly increased after the second sampling and continued to rise during the third and fourth samplings.

Vegetative and reproductive traits were collected for plants grown in both treatments. Plants in the DRT treatment had a final height reduction in 40% compared to the WW treatment, indicating the severity of the stress (Table 1). Plants in the DRT treatment produced 1–2 fewer leaves than WW plants (Table 1). Leaf appearance rate was also slightly delayed under DRT (Table 1 and Supporting Information Figure S2A). Plants flowered (pollen shedding and silk emergence) on the same day (July 10th) in the WW plots. In contrast, plants in the DRT treatment shed pollen 3 days after the WW plots and no silk exertion was observed. DRT treatment ears were dissected at the end of the experiment (July 10th) and showed minimal elongated silks compared to WW ears (Figure 1d,e), resulting in ears with no kernel set.

**Table 1:** Vegetative and reproductive traits of B73 plants in the field, Woodland, CA

Treatment	Plant height (cm)	Leaf No.	Leaf appearance rate (leaves/day)	GDDc to shed	GDDc to silk	Tassel length (cm)	Ear length (cm) by GDDc			Ear elongation rate (cm/day)	Sp ikelet no. at 836 GDDc
							719	792	836		
WW	249.4 ± 8.2	21.1 ± 0.6	0.34	790 ± 4.9	790 ± 4.9	33.3 ± 3.6	3.8 ± 0.6	7.2 ± 0.8	8.5 ± 0.6	0.47	48.3 ± 2.6
DRT	147.8 ± 10.9^a^	18.9 ± 2.0^a^	0.29	832 ± 6.2^a^	No Obs.	26.6 ± 3.8^a^	2.9 ± 0.5^a^	3.7 ± 0.7^a^	6.0 ± 1.2^a^	0.29	47.6 ± 3.5

Height and leaf number were collected from 28 well‐watered (WW) and 32 drought (DRT) treated plants at GDD_C_ 836. Staging notes were taken from V7 to R1. Tassel length was measured for 11 (WW) and 16 (DRT) plants at R1. Leaf appearance rate and ear elongation rate was calculated using linear regression based on data in Supporting Information Figure S2. Measurements represent mean ± *SD*. One day is approx. 11.4 GDD_C_, growing degree days.

a DRT means are statistically different from WW at *p* < 0.01.

The length of the main tassel spike was reduced by ~20% in DRT plants compared to WW plants (Table 1). The ear elongation rate was reduced approximately 1.6‐fold under DRT relative to WW, which resulted in 30% smaller ears under stress (Table 1 and Supporting Information Figure S2B). Remarkably, the final spikelet number per row was not statistically different between treatments. This observation suggests that ear elongation is more susceptible to drought stress than total spikelet number.

To further substantiate this observation, a precision phenotyping experiment was executed using a field pot approach (Supporting Information Figure S4) in Johnston, IA during the summer of 2013. In general, the weather was hot and dry with little precipitation during the experiment (Supporting Information Figure S3). The DRT treatment was initiated when the plants reached the V7–V8 stage, similar to the Woodland experiment. When 50% of plants in the DRT treatment showed obvious signs of leaf wilting, pots were re‐watered to full soil capacity to keep the plants from dying. This dry‐down/rehydration cycle was sequentially repeated eight times from growth stage V8 through R3.

**Figure 2:**
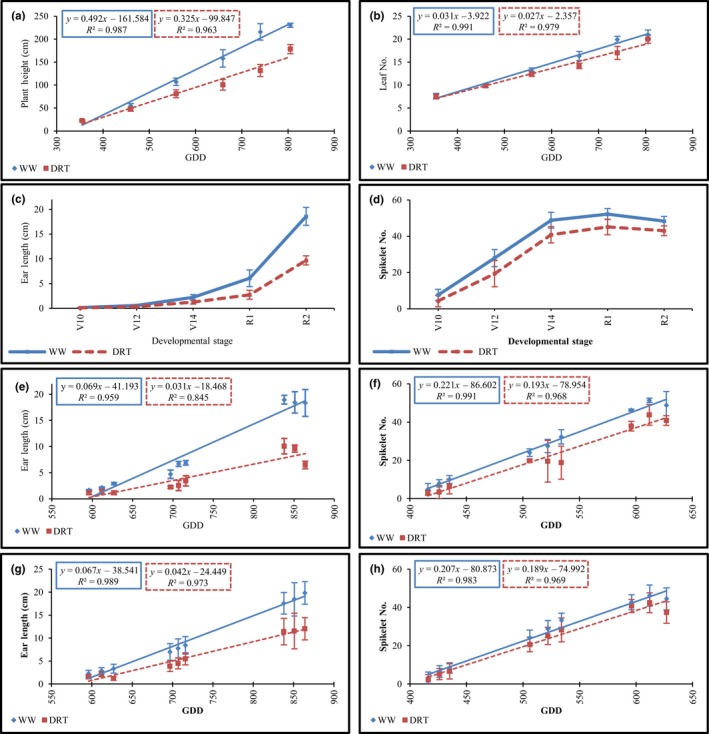
Plant growth and development under WW and DRT in the field pot study, Johnston IA, 2013. Effect of water treatment on B73 plant growth (a) and leaf appearance (b) by GDD_C_ depicted as linear trend lines. Effect of water treatment on ear length (c) and spikelet number (d) by developmental stage. Linear trend lines of ear elongation after V14 (e) and spikelet initiation before V14 (f) by GDD_C_ in B73. Effect of water treatment on average ear elongation (g) and spikelet initiation (h) in nine pioneer proprietary inbred lines by GDD_C_. Trend lines were calculated using linear regression models for each trait and water treatment. The x value in the linear regression formula denotes average change in each trait per 1 GDD_C_. R^2^ describes how well the data fit the trend line where 1.0 is a perfect fit. Data points represent means ± *SD*. Traits separated by WW (solid blue) and DRT (dashed red) treatments

Plant height and vegetative stages were recorded weekly. The plant growth rate, estimated by linear regression models, was decreased 34% in DRT plants compared to WW plants, resulting in a 20% reduction in final plant height relative to the WW treatment (Table 2 and Figure 2a). The estimated leaf appearance rate was only slightly reduced under DRT (Table 2 and Figure 2b). There was no significant difference in final leaf number between DRT and WW treatments (Table 2). However, there was a significant increase in the anthesis silking interval (ASI) in the DRT treatment compared to WW (Table 2).

**Table 2:** Vegetative and flowering traits of B73 in the field pot study, Johnston, IA

Treatment	Plant height (cm)	Plant growth rate (cm/day)	Leaf no.	Leaf appearance rate (leaves/day)	GDD_C_ to shed	GDD_C_ to silk	ASI
WW	223.9 ± 12.8	5.41	20.4 ± 0.8	0.34	715.9 ± 14.5	730.5 ± 14.0	1.6 ± 1.1
DRT	178.4 ± 9.9^a^	3.57	20.0 ± 0.9	0.29	750.5 ± 13.5^a^	798.4 ± 11.0^a^	4.8 ± 1.9^a^

Vegetative traits were collected weekly. Final measurements were collected at R2 stage. Growth rate and leaf appearance rate were calculated using linear regression based on data in Figure 2. Measurements represent mean ± *SD*. One day is approx. 11 GDD_C_, growing degree days.

a DRT means are statistically different from WW at *p* < 0.01.

To investigate ear growth and development, primary ears were dissected at five developmental stages starting at V10 (Supporting Information Figure S5). Ear length and spikelet number (measured as the number of spikelets along the length of the ear) were recorded for each sampled ear (Figure 2c,d). Ear elongation rate after V14, as estimated by linear regression models, was decreased by twofold in DRT relative to WW (Table 3 and Figure 2e) which resulted in 50% shorter ears under DRT (Table 3 and Supporting Information Figure S6). This was similar to the reduction in ear length observed in the Woodland experiment (Table 1 and Supporting Information Figure S2).

**Table 3:** Female inflorescence traits of B73 in the field pot study, Johnston, IA

Treatment	Ear length (cm) by Stage					Ear elongation rate (cm/day)	Max. no. spikelets	Spikelet initiation rate (spikelets/day)
	V10	V12	V14	R1	R2			
WW	0.1 ± 0.06	0.6 ± 0.1	2.2 ± 0.6	6.1 ± 1.7	18.6 ± 1.8	0.76	52.2 ± 3.0	2.43
DRT	0.1 ± 0.05	0.4 ± 0.2	1.2 ± 0.4^a^	2.7 ± 0.9^a^	9.7 ± 0.9^a^	0.35	45.1 ± 4.3^a^	2.13

Ear elongation rate was calculated using data collected after V14 (GDD_C_ 600). Spikelet initiation rate was calculated using data collected prior to V14 (GDD_C_ 600). The maximum number of spikelets occurred at R1 (GDD_C_ 750). Spikelet initiation rate and ear elongation rate were calculated using linear regression based on data in Figure 2. Measurements represent mean ± *SD*. One day is approx. 11 GDD_C_, growing degree days.

a DRT means are statistically different from WW at *p* < 0.01.

Spikelet initiation plateaued around V14 under both conditions (Figure 2d). The estimated spikelet initiation rate before V14 was slightly lower under DRT, 2.13 spikelets per day, compared to 2.43 spikelets per day for WW (Table 3 and Figure 2f). The maximum spikelet number was reduced by 13% under DRT, relative to WW, while ear length was reduced by 50%. This is consistent with the 2012 study in Woodland. To investigate whether the spikelet number is less susceptible than ear elongation to stress in germplasm other than B73, we measured nine Pioneer proprietary inbred lines in the 2013 experiment. The results confirmed that, in general, ear elongation is more susceptible to DRT than ear spikelet number (Figure 2g,h).

Overall, the B73 response to drought stress in the field pot study was comparable to the Woodland field experiment, which suggested that the level of stress was similar in both field experiments. The DRT treatment began at the same developmental stages in both locations and the weather was fairly consistent over the course of both experiments. Data from both studies demonstrated that overall plant growth was more sensitive to drought than progression through developmental stages. The same pattern was observed for the ear, where length was more affected by drought than spikelet initiation.

### 3.2 Analysis of RNA‐seq expression in leaf, ear, and tassel samples

To conduct RNA‐seq transcription profiling, leaves, ears, and tassels were systematically sampled after initiation of the DRT treatment in the Woodland experiment (Supporting Information Figure S1). At the first sampling plants were at V12 (ears < 0.5 cm), followed by a second sampling at V14 (ears 0.8–1.5 cm). The third sampling point was at V18 (ears 3–4 cm under WW), followed by a final sampling at R1 (ears 7–8 cm under WW). No silk emerged under DRT conditions even though it was formed inside of the husk. Ninety‐six RNA–seq libraries were generated and were comprised of three tissues, four sampling times, four biological replicates, and the two treatments (WW and DRT). Quality control and mapping reads to the B73 reference genome are described in a recent publication (Thatcher et al., 2016). The total number of expressed genes was in the range of 31,000–37,000 by tissue (Supporting Information Figure S7). The number of DE genes varied substantially by tissue, with leaves having the fewest (~3,500), followed by ear (~7,000), and finally tassel (~20,000) (Supporting Information Figure S7).

**Figure 3:**
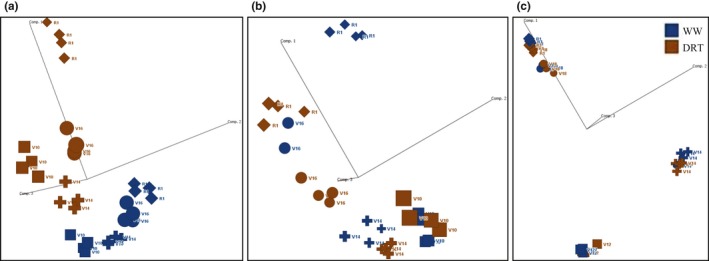
Principal component analysis of differentially expressed (DE) genes. Principal components were calculated based on the expression levels of genes across tissues (component 1), developmental stages (component 2), and treatment (component 3). Leaf samples include 3,454 DE genes (a), ear samples include 6,946 DE genes (b), tassel tissue samples 19,850 DE genes (c). Blue color represents WW samples; brown color represents DRT samples. The following shapes represent plant stages at sampling: square ‐ V12, cross –V14, circle – V18, diamond –R1. There are four replicated samples for each stage

Principal component analysis was used to identify the major experimental factors (components) accounting for all DE genes across tissues, developmental stages, and treatments. Leaf WW samples were primarily separated from DRT samples based on water treatment rather than development stage, suggesting that the majority of DE genes were responding to the drought treatment in mature segments of the leaf (Figure 3a). In contrast, WW and DRT ear samples were primarily clustered by development stages rather than by water treatment, reflecting developmental processes occurring in the ears (Figure 3b). At the later stages, a treatment specific clustering began to emerge. This suggests that most DE genes in ears are initially driven by development, but begin to respond to treatment over time (Figure 3b). Virtually no treatment effect was detected in the tassel samples (Figure 3c) suggesting that most DE genes were related to development rather than a result of watering treatment.

In order to identify DE genes responding to drought, K‐Means clustering was performed at every sampling stage for WW and DRT samples of each tissue with a Q‐value cutoff of 1E‐2 from the set of DE genes (Supporting Information Figure S8). K‐Means clustering was well supported by four biological replicates per sampling (Supporting Information Figures S9‐S11). The clustering was then used to populate Gene Ontology (GO) categories.

**Figure 4:**
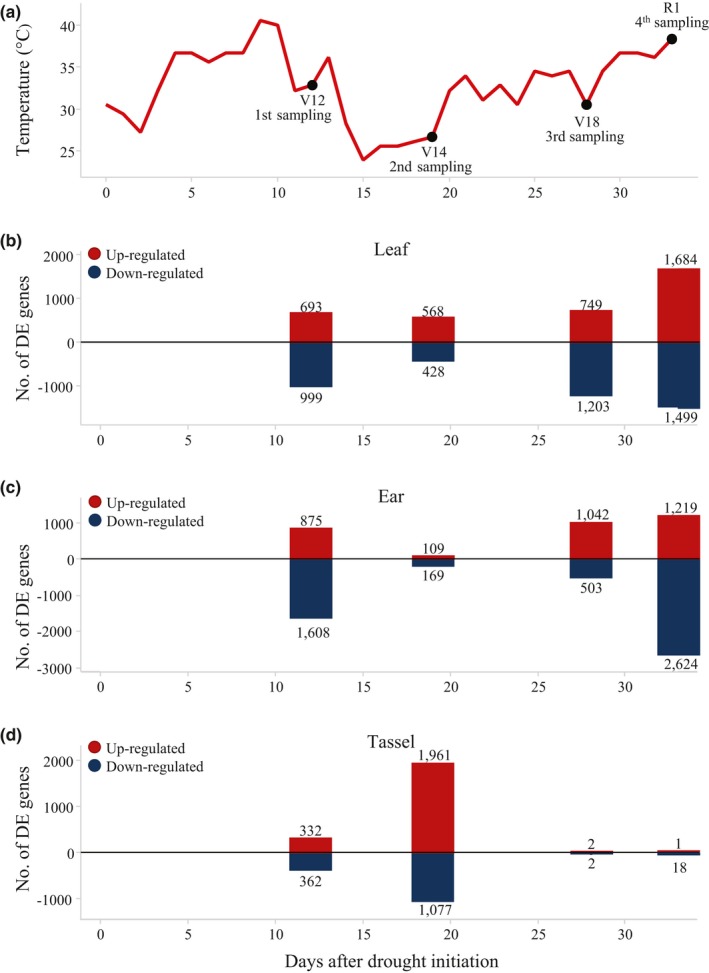
Distribution of DE genes in vegetative and reproductive tissue by sampling times. Daily temperature (°C) patterns during the experiment sampling days and plants V‐stages are marked by dots. First sampling was done at 11 days of drought stress, second sampling at 18 days of drought, third sampling at 27 days of drought, fourth sampling at 32 days of drought (a). Distribution of DRT up‐regulated (red) and DRT down‐regulated (blue) genes in leaves (b), ears (c), and tassels (d)

It is important to consider the weather pattern during the Woodland field experiment, which imposed heat as an additional stress factor to drought (Figure 4a). During the first week of drought stress, daily temperatures were 35⁰C to 40⁰C, exposing plants at the V12 sampling to not only drought stress, but also heat stress. The week before the V14 sampling, daily temperatures were 25⁰C and plants experienced drought stress but less heat stress. The daily temperature then progressively increased from 30°C to 40°C, suggesting the highest level of combined heat and drought stress occurred after 32 days of drought stress at the R1sampling date.

Every tissue displays unique changes at the transcriptional level in response to abiotic stress. Leaf transcriptional changes increased, in general, over the course of the drought treatment, reaching the maximum number of DE genes at 32 days of drought (R1) (Figure 4b). At V14 when the temperature was mild, the number of DE genes was slightly reduced relative to V12 and V18 samplings (Figure 4b). In contrast to the other samplings, at R1 more genes were up‐regulated than down‐regulated in leaves.

The ear transcriptional response displayed a more pronounced bimodal pattern than the response in leaves (Figure 4c). The largest number of DE genes was detected at the V12 and R1 samplings. At the V14 sampling the number of DE genes dropped ~10‐fold (Figure 4c) from the V12 stage. Then DE genes increased continually from the V18 sampling to reach the maximum differential expression at the R1 sampling (Figure 4c). The dynamics of the DE gene response was correlated with the weather pattern; when drought and heat stress were at a maximum, more DE genes were observed. There were more down‐regulated relative to up‐regulated genes at the later stages. At the R1 sampling, when the average daily temperature once again reached 40°C, the number of down‐regulated genes increased by ~5‐fold, whereas the number of up‐regulated genes was similar to the previous V18 sampling (Figure 4c). Tassel had the lowest transcriptional responses to stress and showed a pattern opposite to that of the ear. The maximum numbers of DE genes were detected in the tassel at the V14 stage when ears had the smallest number of DE genes (Figure 4c,d). The heat stress level was the lowest at this time. However, at the highest level of drought and heat stress (third and fourth samplings) few DE genes were found in tassel samples. This pattern suggests that the tassel transcriptome is less responsive to environmental stress which agrees with the phenotypic observation that tassel development is less sensitive to drought stress relative to ear development.

### 3.3 Gene ontology enrichment of biological processes for de genes in leaf samples

**Figure 5:**
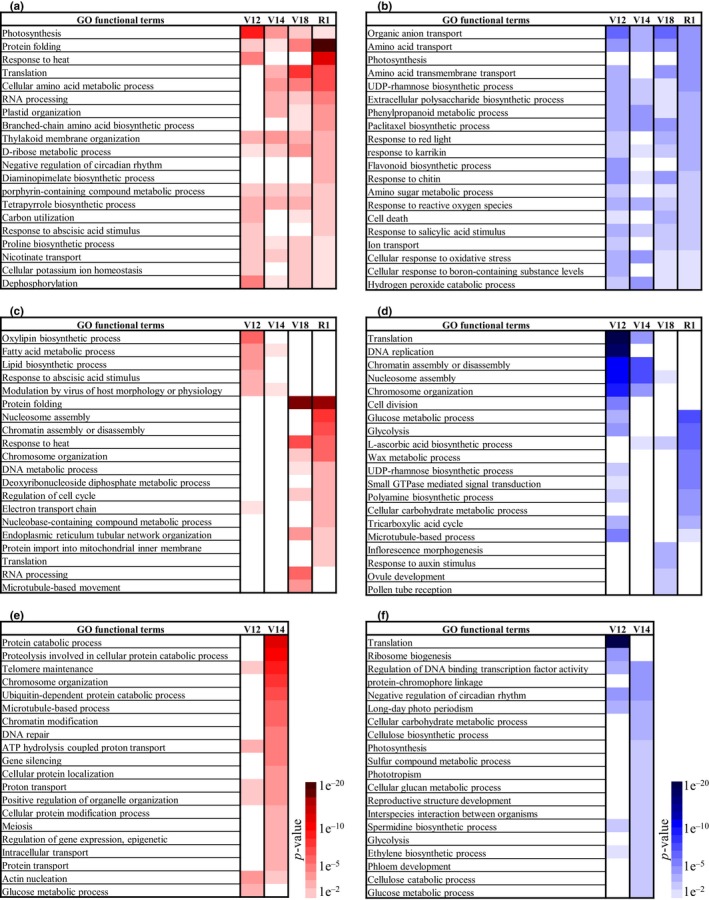
Gene Ontology (GO) enrichment of biological processes for DE genes under drought stress. Gene Ontology Fisher's exact test was used for the enrichment analysis of the DE genes (Table 4). A *p*‐value of 0.01 was used to select enriched GO terms. *p*‐value for drought stress up‐regulated terms shown in red, and down‐regulated terms shown in blue. The lowest *p*‐values are depicted by darker color. a and b leaves, c and d ears, e and f – tassels

In order to gain insight into the functional categories of drought responsive genes, gene ontology (GO) Fisher's exact test was used to analyze DE genes separated by tissue and stage. The tissue‐specific top 20 up‐ and down‐regulated GO functional terms, excluding high‐level and synonym terms, are presented in Figure 5. The complete dataset is available in Supporting Information Table S1 (leaf), Table S2 (ear), and Table S3 (tassel).

#### 3.3.1 Leaf up‐regulated genes

A consistent enrichment across developmental stages was observed in the “protein folding” category, reaching a maximum at the R1 sampling (Figure 5a). Genes in the “response to heat” category were enriched in the V12 and R1 samplings which followed the temperature pattern. Gene enrichments in “cellular amino acid metabolic process” and “proline biosynthetic process” categories were also enriched, suggesting enhancement of osmolyte biosynthesis, such as proline and other amino acids. Some enrichment was also observed in genes related to translation machinery, suggesting stabilization of protein biosynthesis under drought conditions.

Surprisingly, there was enrichment of up‐regulated genes in functional categories related to photosynthetic machinery such as “photosynthesis light reaction and light harvesting”, “electron transport in photosystem II”, “chlorophyll biosynthetic process”, “porphyrin‐containing compound metabolic process”, “tetrapyrrole biosynthetic process”, “phylloquinone biosynthetic process”, and “thylakoid membrane organization” (Supporting Information Table S1). At later stages, the level of enrichment gradually declined (Figure 5a). This suggests that there may be compensatory responses in leaves at the first sign of drought stress to maintain photosynthesis. As drought stress continues and less water is available, leaves begin to show daytime wilting (Figure 1b) which likely impedes the photosynthetic rate. A similar up‐regulation of photosynthetic genes under drought stress in B73 leaves was recently reported (Avramova et al., 2015). However, in that study, photosynthesis was inhibited by stress as well as stomata conductance. To reconcile this paradox, the authors provided some evidence that investment in the photosynthetic machinery under stress facilitates photosynthesis during plant recovery when water becomes available (Avramova et al., 2015).

Abscisic acid (ABA) is a primary abiotic stress response hormone and is expected to have a strong response to drought (Hauser, Waadt, & Schroeder, 2011). However, few genes were identified in the “cellular response to abscisic acid (ABA) stimulus” category. The highest level of drought response was shown by the bZIP transcription factor *ABI‐5* (GRMZM2G479760, ABSCISIC ACID‐INSENSITIVE‐5) and duplicated genes (GRMZM2G073324 and GRMZM2G389301, EID1‐like F‐box protein 3), which are homologs of the Arabidopsis gene *EDL3* involved in the regulation of ABA‐signaling (Koops et al., 2011). The ABA biosynthesis gene *VP14* (GRMZM2G014392 viviparous14, 9‐cis‐epoxycarotenoid, NCED1 dioxygenase) was also up‐regulated at the V12 sampling.

#### 3.3.2 Leaf down‐regulated genes

The greatest number of down‐regulated genes was found in the “organic anion transport” and “amino acid transport” categories (Figure 5b). This indicates that with low water movement through the plant under DRT conditions a reduction in transport of solutes may occur. Down‐regulation of genes in biological categories such as “response to chitin” and “salicylic acid stimulus” suggests that plant immunity to pathogen invasion may weaken under drought conditions. The trend of down‐regulation of genes involved in the “response to reactive oxygen species” was also observed, which could cause reactive oxygen species to accumulate in leaves under drought stress.

### 3.4 GO enrichment of biological processes for DE genes in ear samples

#### 3.4.1 Ear up‐regulated genes

The bimodal distribution of DE genes in ear tissue was paralleled by the diverse functions at the beginning and end of the abiotic stress period. The early responsive genes at the V12 sampling were enriched in the “oxylipin biosynthetic processes” category (Figure 5c). One important example of oxylipins is the biotic stress hormone jasmonic acid (JA) (Wasternack & Hause, 2013). The JA precursor, 12‐oxo‐phytodienoic acid (12‐OPDA), is also a biologically active molecule having similar function to JA (Savchenko, Zastrijnaja, & Klimov, 2014). The individual genes in the oxylipin category are positioned at the upstream steps in the JA biosynthetic pathway. These genes include *LOX1* (GRMZM2G156861, Lipoxygenase 1), *LOX3* (GRMZM2G109130), *LOX6* (GRMZM2G040095), and *AOS* (GRMZM2G067225, allene oxide synthase) suggesting that oxylipin biosynthesis may be up‐regulated in ears under stress. Moreover, the JA receptor *COI* (coronatine‐insensitive) genes (GRMZM2G125411, GRMZM2G353209, GRMZM2G151536) were also up‐regulated, suggesting enhanced signaling for JA and other oxylipins.

In the “response to abscisic acid stimulus” category, duplicated genes *EDL3* (GRMZM2G073324 and GRMZM2G389301 EID1‐like F‐box protein 3), bZIP transcription factor *ABI‐5* (GRMZM2G077124), and the ABA receptor *PYL8* gene (GRMZM2G165567) were all up‐regulated. This is similar to what was observed in leaves, except that in leaves activation of the ABA receptor genes was not detected.

No enrichment of the typical stress response categories such as “protein folding” or “response to heat” were found in ear samples at the V12 or V14 samplings. However, these categories were enriched later at the V18 and R1 samplings when plants had been exposed to abiotic stress for a longer period of time. At the later stages, some enrichment of up‐regulated genes was observed in the “cell cycle” and “DNA replication” categories which may be explained by delayed ear growth under drought conditions (Figure 1d,e and Supporting Information Figure S2B).

#### 3.4.2 Ear down‐regulated genes

The categories with the greatest number of down‐regulated genes in ears were “translation”, “DNA replication”, “cell division”, and many related processes (Figure 5d and Supporting Information Table S2). These down‐regulated genes were predominant at the V12 sampling, but were less impacted at later stages. These results suggest that fundamental processes such as protein biosynthesis, DNA replication and cell division are suppressed under a combination of heat and drought stress, resulting in a delay in ear growth. As soon as environmental conditions were less stressful (V14 sampling), transcriptional activity of these key genes was restored. Down‐regulation of genes in the categories of “glucose metabolic process”, “glycolysis”, and “tricarboxylic acid cycle” was observed at the V12 and R1 samplings, indicating the sensitivity of energy related processes to stress conditions. Down‐regulation of genes involved in carbohydrate metabolic processes was enriched at the R1 stage. Interestingly, genes in the “wax metabolic processes” were down‐regulated (R1 stage) signifying that wax biosynthesis may be impaired.

### 3.5 GO enrichment of biological processes for DE genes in tassel samples

In contrast to the ear and leaf, drought response in the tassel was much more limited (Figure 4b,c,d). The majority of the DE genes were seen at the V14 sampling. The most prominent enrichment among the up‐regulated genes was found in processes related to “protein degradation”, “telomere maintenance”, “chromosome and chromatin organization”, and “meiosis” (Figure 5e). Among the down‐regulated genes, functional enrichments were observed in “translation” and “ribosome biogenesis” categories (Figure 5f). At the later V18 and R1 stages there were very few differences in gene expression between WW and DRT samples. Transcriptional differences appeared to reflect a slight delay in tassel development under DRT and not a direct effect of stress on the tassel transcriptome.

### 3.6 Comparison of top ranking stress‐induced categories in leaf and ear samples

**Figure 6:**
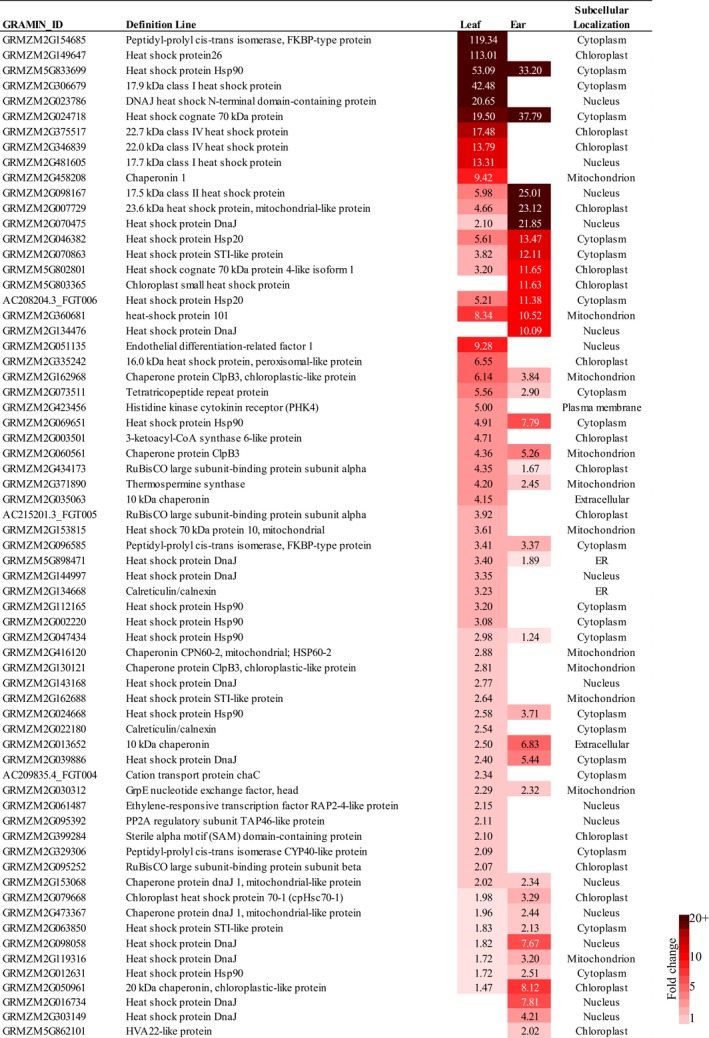
Expression of genes in categories “protein folding” and “heat response” in leaves and ears at the R1 stage. The darkest color corresponds to the higher levels of induction under stress. Cutoff effective size was above twofold threshold at least in one tissue. A default of 10^−6^ FDR was used

Two functional categories “protein folding” and “heat response” were highly enriched in leaves and ears under prolonged drought stress (Figure 5a,c). Neither of these functional categories was enriched in the tassel. We detected 66 genes whose expression level was increased >twofold in at least one tissue at the R1 stage (Figure 6). The majority of these were annotated as heat‐shock proteins (HSP). There were 61 HSP genes that were up‐regulated in leaves and 36 HSP genes in ears. The subcellular localization of the predicted proteins suggested that about 17 of them are localized to the chloroplast, signifying their putative function in protecting the photosynthetic machinery. In leaves, the top up‐regulated genes encode the peptidyl‐prolyl cis‐trans isomerase (GRMZM2G15468) and HSP26 (GRMZM2G149647) were induced by stress >110‐fold. However, these genes were not expressed in ears. The top DRT induced genes in ears were the heat shock gene *HSP90* (GRMZM5G833699) and *HSP70* (GRMZM2G024718). These genes were induced 33‐ and 38‐fold, respectively in ears and by 53‐ and 20‐fold, respectively in leaves.

### 3.7 Expression of developmental genes in stressed ears

**Figure 7:**
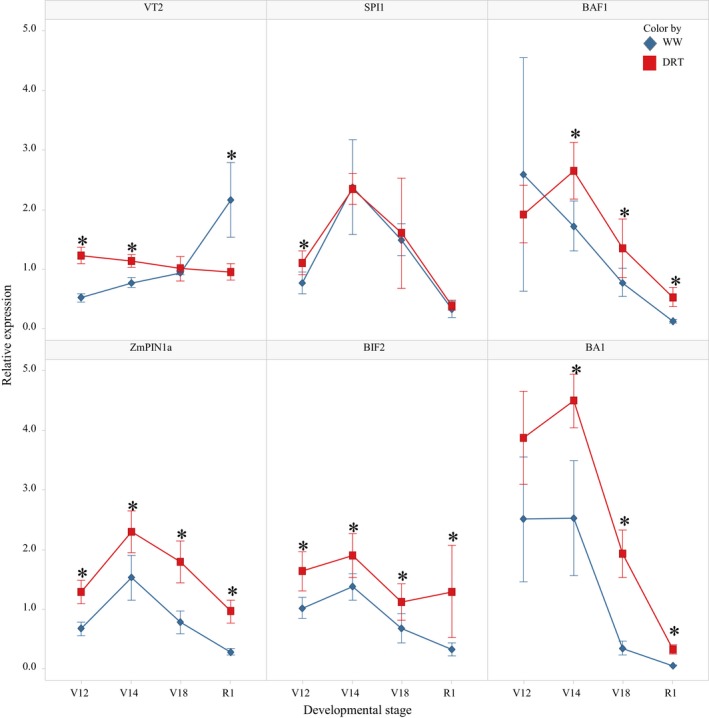
Expression of axillary meristem initiation genes in developing ears (qRT‐PCR). *DRT means are statistically different from WW at *p* < 0.01

It is important to note that there were few differentially expressed inflorescence development genes identified in stressed ears, even though the entire meristem spectrum (from IM to SPM to SM and FM) was sampled over the course of the experiment. Some degree of enrichment was observed among down‐regulated genes at the V14 stage in the “inflorescence morphogenesis” category (Figure 5d) and at the R1 stage in the “sexual reproduction”, “cell morphogenesis”, and “developmental process involved in reproduction” categories (Supporting Information Table S2). The small number of developmental genes identified in the stressed ear samples led us to conduct qRT‐PCR of several characterized maize genes involved in the patterning of the maize inflorescences (Table 4). We tested six genes essential for axillary meristem initiation: *VT2* (*vanishing tassel2*) (Phillips et al., 2011), *SPI1* (*sparse inflorescence1*) (Gallavotti, Barazesh, et al., 2008), *ZmPIN1a* (*pinformed*) (Gallavotti, Yang, Schmidt, & Jackson, 2008), *BAF1* (*barren stalk fastigiated*) (Gallavotti et al., 2011), *BIF2* (*barren inflorescence2*) (McSteen et al., 2007), and *BA1* (*barren stalk1*) (Gallavotti et al., 2004). The expression patterns of these genes were similar in WW and DRT ear samples (Figure 7). None were repressed by stress conditions and several were slightly up‐regulated with a *p*‐value of 0.01. Absence of repression under stress conditions is consistent with the phenotypic data that show a limited effect of stress on spikelet initiation and spikelet number.

**Table 4:** Expression of maize developmental genes in ears under drought in the field, Woodland, CA

GRMZM_ID	Gene name	Protein	Response to stress
Axillary meristem initiation			
GRMZM2G127308	*VT2 (vanishing tassel2)*	Tryptophan Aminotransferase	Neutral^a^
GRMZM2G025222	*SPI1 (sparse inflorescence1)*	Flavin auxin oxygenase	Neutral^a^
GRMZM2G098643	*ZmPINa (pinformed)*	Auxin efflux transporter	Neutral^a^
GRMZM2G072274	*BAF1 (barren stalk fastigiated)*	AT‐hook TF	Neutral^a^
GRMZM2G171822	*BIF2 (barren inflorescence2)*	Protein kinase	Neutral^a^
GRMZM2G397518	*BA1 (barren stalk1)*	bHLH TF	Neutral^a^
Inflorescence meristem size			
GRMZM2G017087	*KN1 (knotted1)*	Homeodomain TF	Neutral^a^
GRMZM2G104925	*FEA2 (fasciated ear2)*	CLV2‐like receptor	Neutral^a^
GRMZM2G133331	*FEA4 (fasciated ear4)*	bZIP TF	Neutral^a^
GRMZM2G300133	*TD1 (thick tassel dwarf1)*	CLV1‐like receptor	Neutral
Inflorescence meristem determinacy			
GRMZM2G003927	*RA1 (ramosa1)*	Zinc finger TF	Neutral^a^
AC233943.1_FGT002	*RA2 (ramosa2)*	LOB‐domain TF	Neutral^a^
GRMZM2G014729	*RA3 (ramosa3)*	T‐6‐P phosphatase	Neutral^a^
GRMZM2G042992	*REL2 (ramosa1 enhancer locus2)*	TOPLESS TF co‐repressor	Down
GRMZM2G307119	*BD1 (branched silkless1)*	Ethylene‐responsive TF	Neutral^a^
Floral development			
GRMZM2G148693	*ZAP1 (Zea apetala homolog1)*	MADS TF, AP‐FUL clade, class A	Neutral^a^
GRMZM2G110153	*ZMM16 (Zea mays MADS16)*	MADS TF, GLO clade, class B	Down
GRMZM2G139073	*SI1 (silky1)*	MADS TF, DEF clade, class B	Neutral^a^
GRMZM2G052890	*ZAG1 (Zea AGAMOUS homolog1)*	MADS TF, AG clade, class C‐D	Down^a^
GRMZM2G160565	*ZAG3/BDE1 (bearded‐ear1)*	MADS TF, AGL6 clade	Down^a^
GRMZM2G160687	*ZAG2 (Zea AGAMOUS homolog2)*	MADS TF, AG clade, class C‐D	Down^a^
GRMZM2G359952	*ZMM2 (Zea mays MADS2)*	MADS TF, AG clade, class C‐D	Down^a^
GRMZM2G471089	*ZMM23 (Zea mays MADS23)*	MADS TF, AG clade, class C‐D	Down^a^
GRMZM2G003514	*ZAG5 (Zea agamous5)*	MADS TF, AGL6 clade	Neutral^a^
GRMZM2G087095	*ZMM24 (Zea mays MADS24)*	MADS TF, SEP clade, class E	Down
GRMZM2G071620	*ZMM31 (Zea mays MADS31)*	MADS TF, SEP clade, class E	Down
GRMZM2G159397	*ZMM6 (Zea mays MADS6)*	MADS TF, SEP clade, class E	Down
GRMZM2G099522	*ZMM14 (Zea mays MADS14)*	MADS TF, SEP clade, class E	Down
GRMZM2G105387	*MADS TF‐box26*	MADS TF, AGL12 clade	Down
GRMZM2G117961	*MADS TF‐box26*	MADS TF, AGL12 clade	Down
GRMZM2G005155	*MADS TF‐box1*	MADS TF	Down
GRMZM2G018589	*MADS TF‐box58*	MADS TF, AG clade, class C‐D	Down
GRMZM2G097059	*MADS TF‐box7*	MADS TF, SEP clade, class E	Down
GRMZM5G862109	*TS6 (tasselseed6)*	APETALA2‐like TF	Down
GRMZM2G076602	*AP2 (floral homeotic protein)*	APETALA2‐like TF	Down
GRMZM2G102218	*DL (drooping leaf homolog)*	YABBY TF	Down
Embryo sac development			
GRMZM2G118250	*IG1 (indeterminate gametophyte1)*	LOB‐domain TF	Down
GRMZM2G042055	*FERONIA homolog*	Receptor kinase	Down

Response to stress is shown as a trend based on RNA‐seq expression. Genes are grouped into functional categories. TF stands for Transcription Factor.

a Denotes expression validated by qRT‐PCR. Other genes were not tested.

We also tested selected genes involved in IM maintenance and size such as *KN1 (homeotic protein knotted1)*,* FEA2* (*fasciated ear2*), and *FEA4* (*fasciated ear4*) (Pautler et al., 2015). There was no significant effect of stress on expression patterns of these genes (Supporting Information Figure S12). The meristem determinacy genes *RA1, RA2*, and *RA3 (Ramosa1, 2, and 3)* (Tanaka et al., 2013) as well as *BD1* (*branched silkless1*) (Chuck, Muszynski, Kellogg, Hake, & Schmidt, 2002) showed only minor responses to stress (Supporting Information Figure S12).

We also investigated expression of MADS box transcription factors which are key regulators of floral development. Out of the 43 maize MIKC‐type MADS box genes (Zhao et al., 2010), at least 15 genes were down‐regulated in ears under stress (Table 4). Expression patterns were confirmed by qRT‐PCR for eight selected MADS box genes (Supporting Information Figure S13). Non‐MADS floral developmental genes such as *TS6* (*tasselseed6*), *AP2* (*floral homeotic*), and *DL* (*drooping leaf* homolog) were also down‐regulated by stress (Table 4). These results suggest that drought stress may delay floral development due to the repression of floral developmental genes.

Down‐regulation of genes related to ovule development and pollen tube receptivity were also found in this study (Table 4). Among them were *IG1* (*indeterminate gametophyte1*) (Evans, 2007) and a homolog of the Arabidopsis *FERONIA* gene. FERONIA is receptor‐like protein kinase that is essential for interaction between the synergids and the pollen tube during embryo sac fertilization (Escobar‐Restrepo et al., 2007). Another function of FERONIA is the regulation of cell elongation (Haruta, Sabat, Stecker, Minkoff, & Sussman, 2014). Down‐regulation of genes involved in embryo sac development suggests a negative effect of abiotic stress on the formation of functional ovaries.

## 4 DISCUSSION

### 4.1 Plant growth and development is affected by stress in a tissue specific manner

Drought stress is an area of extensive research in many crops and model plants because of its negative impact on agriculture productivity (Blum, 2014). It has been well documented that the failure of the maize female inflorescence (ear) to develop under stress conditions is a primary reason for grain yield loss in the field (Araus et al., 2012; Barker et al., 2005; Boyer & Westgate, 2004; Campos et al., 2006). Previous genomic studies of maize reproductive tissues grown under water‐limiting conditions provide insight into their general drought response, but fail to comprehensively correlate the maize ear development with transcriptome response to drought under field conditions.

We conducted experiments covering the sequence of ear development starting from initiation up to silk emergence under WW and DRT conditions. This sampling was done in the field to capture ear responses to drought under natural weather conditions. In addition, we systematically collected tassel and leaf samples as well as phenotypic traits for the comparison of tissue specific growth and developmental processes under drought stress.

In both field experiments, plants were exposed to significant drought stress. There were differences in irrigation and high temperature fluctuations between the two locations which may explain some of the small observed phenotypic differences. For example, in Woodland, plant height in the DRT treatment was reduced by 40% relative to the WW treatment, whereas in Johnston, plant height was reduced by 20% suggesting a more severe abiotic stress in Woodland, where on average higher daily temperatures occurred. The leaf number of DRT plants was reduced by 1–2 leaves in Woodland. In Johnston the leaf number was the same at WW and DRT. There were similar leaf appearance rates for WW and DRT plants in both experiments. Overall, this suggests that drought has less impact on developmental stages than on plant growth.

Ear growth appears to be particularly sensitive to abiotic stress relative to ear organogenesis. The rate of ear elongation was twofold slower under the DRT (0.3 cm/day) treatment than the WW treatment (0.5–0.7 cm/day). In addition, DRT ears at silking were approximately 50% shorter than WW ears in both experiments. However, the final number of spikelets per ear row was not significantly different between treatments in the Woodland experiment and was slightly reduced by drought in the Johnston study. This observation in B73 was confirmed by assessing ear traits for nine proprietary Pioneer inbred lines grown in the field pots. All of these inbreds showed a similar number of spikelets under WW and DRT conditions, but a stronger reduction in ear length under drought (Figure 2g,h).

Tassel growth (measured by final tassel length) is less sensitive to abiotic stress than ear growth, being reduced by 20% under drought stress relative to well water as compared to a 50% reduction in ear size under the same conditions (Table 1). This finding is consistent with previously published studies that tassels are less susceptible to drought than ears (Herrero & Johnson, 1980a). Overall, our phenotypic data demonstrate that maize organs have a differential response to abiotic stress. Ear growth is the most sensitive to stress, followed by plant growth (height) and tassel growth (length). Moreover, developmental processes such as leaf and spikelet initiation (organogenesis) are less affected by drought than organ growth, a pattern that matches our transcriptional analysis.

### 4.2 Transcriptome response is tissue specific and adjusts to environmental conditions

Organ specific drought phenology is mirrored by different transcriptome responses in respect to DE gene numbers, functional categories, and stage distribution. In the leaf, the number of DE genes increased during the course of the DRT treatment, reaching a maximum at the R1 stage. Some decline in DE genes was observed at the V14 stage when plants were exposed to more moderate temperatures, suggesting that the transcriptome is responsive to both drought and temperature.

Transcriptional responses to stress in ears were not as evenly distributed across developmental stages. The highest numbers of DE genes were detected in the V12 and R1 samplings when the level of stress was the highest due to a combination drought and heat stress. The lowest numbers of DE genes were found at the V14 sampling when the daily temperature was 25°C, an optimal temperature for maize growth (Abendroth et al., 2011), although the plants had not been irrigated for 10 days. This suggests that the ear might be responding more to heat and drought stress than to drought stress alone. Our results are consistent with RNA profiling of sorghum seedlings, where heat stress induced more genes than drought stress and a combination of both treatments induced more genes than each treatment separately (Johnson et al., 2014). However, the individual impacts of heat and drought on gene expression in maize ears require more additional studies with the proper controlled environment to arrive at the similar conclusion.

The smallest numbers of DE genes were found in the tassel, which is in agreement with its comparatively minor phenotypic response to drought compared to the ear. Unlike other organs, DE genes in the tassel peaked at the V14 stage when the level of abiotic stress was the lowest. This may be explained by an asynchrony of gene expression due to a minor delay of tassel development under stress, rather than by drought‐specific responses at this stage. We could speculate that a greater number of DE genes at the V14 tassel correlates with meiotic activity and pollen grain formation at this stage, which is supported by enrichments of GO terms for meiotic function and telomerase genes (Supporting Information Table S3). Few DE genes were detected in the V18 and R1 samples, which were the periods of the most intense abiotic stress. Moreover, no activation of heat shock genes, which are biomarkers for stress responses, was detected in tassels. Our findings are in agreement with previous studies indicating that due to the shoot apical dominance, tassel growth is less sensitive to stress than ear growth (Herrero & Johnson, 1980a). However, heat, not drought is detrimental for pollen viability (Herrero & Johnson, 1980b; Schoper, Lambert, & Vasilas, 1985).

Activation of heat shock proteins (HSP) is a universal stress response in virtually all organisms. These proteins function as molecular chaperons, preventing proteins from misfolding, denaturing, and degrading in order to support vital functions during stress episodes (Al‐Whaibi, 2011). Genes annotated in GO categories “protein folding” and “response to heat” were among the top up‐regulated genes in the leaf and there was a significant overlap between stress‐induced genes found in our study and those found in sorghum seedlings (Johnson et al., 2014), suggesting conserved stress responses in leaves of C4 grasses. Many leaf‐expressed HSP have predictive subcellular localization in chloroplasts which indicates that they may function to protect chloroplasts from stress (Hu et al., 2015). In the ear, heat shock genes were activated two stages later than in leaves, with smaller numbers and lower fold changes. In total, only 30 heat shock genes were induced in ears, and the genes with the largest change were *HSP90* and *HSP70* which are also induced in the leaf under heat stress. Heat shock proteins of this type are thought to be molecular chaperons that stabilize newly synthesized proteins to support their function under unfavorable conditions (Al‐Whaibi, 2011).

One possible interpretation of the different dynamics of heat shock proteins in the leaf versus the ear is related to different tissue functions. The leaf senses and then rapidly responds to the changing environment by the activation of heat shock genes to protect chloroplasts. In contrast, in the ear, it takes a longer time to activate heat shock genes where their function may be related solely to stabilizing proteins under stressful conditions. The stronger response of heat shock proteins in leaves may be explained by the importance of chloroplast maintenance and the threat of water loss through transpiration. No heat shock genes were induced in the tassel, suggesting that it may be insulated from stress better than the other organs.

### 4.3 Retarded ear growth correlates with the down‐regulation of DNA replication and cell‐cycle genes and the up‐regulation of oxylipin biosynthetic genes under stress

Like previous studies (Boyer & Westgate, 2004; Westgate & Grant, 1989) we have shown that ear growth is very sensitive to abiotic stresses. However, limited data are available to explain the molecular mechanisms of the inhibition of ear growth under stress at developmental stages prior to silk emergence. Our RNA‐seq data show that the first stress response in early V12 stage ears is a massive down‐regulation of genes involved in DNA replication, cell cycle, and cell division (Figure 5d). About 140 genes within these categories had a >1.5‐fold decrease. Down‐regulation of DNA replication and cell‐cycle genes ultimately leads to arrested cell division and cessation of ear growth. A similar response was observed in maize leaves whereby both cell division and cell expansion were down‐regulated under DRT in concert with a decrease in cell cycle gene expression (Avramova et al., 2015). In addition, down‐regulation of cell cycle genes was observed in drought stressed ovules, which is thought to be a cause of early kernel abortion (Kakumanu et al., 2012).

At the V12 stage, the greatest number of up‐regulated genes was in the “oxylipin biosynthetic process”, “lipid biosynthetic process”, and “response to abscisic acid stimulus” categories. Activation of ABA‐signaling pathways was expected, but the up‐regulation of the oxylipin pathway appears to be novel. The most studied oxylipin is the plant defense hormone jasmonate, with a broad range of functions in growth, development, biotic (Wasternack & Hause, 2013) and abiotic stress responses (Savchenko, Zastrijnaja, et al., 2014). Upstream genes in the biosynthetic oxylipin pathway *AOS*,* LOX1*,* LOX3, LOX6* and two lipoxygenase genes with no specific names (GRMZM2G015419, GRMZM2G009479) were up‐regulated. Similar drought induced expression of *LOX* genes has been observed in the leaf elongation zone (Avramova et al., 2015). Induction of these genes by drought may lead to an accumulation of 12‐oxo‐phytodienoic acid (12‐OPDA), a precursor of jasmonic acid as was shown in Arabidopsis (Savchenko, Kolla, et al., 2014). The up‐regulation of three maize homologs of the *COI* genes, which encode the jasmonate receptor that plays a key role in JA‐signaling (Wasternack & Hause, 2013), were also found. Jasmonates are known repressors of cell‐cycle genes especially in dividing tissues (Pauwels, Inze, & Goossens, 2009; Pauwels et al., 2008; Shyu & Brutnell, 2015). The activation of jasmonate biosynthesis and signaling pathways in developing ears may lead to repression of DNA replication, cell cycle, and cell division genes resulting in ear growth inhibition. We propose that jasmonates (or their precursors) may be the factors controlling ear growth under abiotic stress conditions which is supported by the emerging function of jasmonates in abiotic stress responses beyond their well‐known roles in biotic stress responses (Kazan,^2015^; Liu et al., 2015).

### 4.4 Spikelet initiation is tolerant to stress which correlates with the stable expression of inflorescence meristem genes

Robust spikelet initiation was observed in ears under abiotic stress conditions. In Woodland, there was no difference between treatments in the final number of spikelets formed on the ear. While in Johnston, the spikelet number was modestly reduced under drought as compared to a dramatic reduction in ear length. Moreover, a study of nine Pioneer inbred lines showed a limited effect of drought on spikelet initiation and final number (Figure 2h). Spikelet number is determined by local auxin biosynthesis and auxin signaling in the inflorescence meristems that produces SPM and SM (Barazesh & McSteen, 2008; Gallavotti, 2013). There are at least six maize genes with well‐established functions in spikelet initiation: *BAF1* is required for ear formation (Gallavotti et al., 2011), *VT2* and *SPI1* function in local auxin biosynthesis (Gallavotti, Barazesh, et al., 2008b; Phillips et al., 2011), and *ZmPIN2a, BIF2,* and *BA1* regulate auxin transport and signaling (Barazesh, Nowbakht, & McSteen, 2009; Gallavotti, Yang, et al., 2008; Gallavotti et al., 2004). Our data show that none of these genes are repressed in ears under drought stress (Figure 7). This finding is consistent with the limited effect of stress on the spikelet number (Table 4). Additionally, no significant expression differences were detected in genes controlling the inflorescence meristem size and maintenance (*KN1, FEA2, 4*,* TD1),* or meristem determinacy (*RA1, 2, 3, BD1)* (Table 4). These findings suggest that the initial steps in ear development are tolerant to abiotic stress. Despite the severity of abiotic stress in this experiment, the DRT stressed ears still generate spikelet numbers similar to that of WW ears. Spikelet initiation may be relatively resistant to abiotic stress in order to keep the possibility of returning to full reproductive competence, if water limited conditions abate.

### 4.5 Down‐regulation of floral development genes is consistent with the formation of defective ovaries under stress

Each spikelet (axillary) meristem (SM) gives rise to a floral meristem (FM) which develops into the gynoecium and terminates in formation of the ovary. The ovary is composed of the embryo sac and silk, a stigmatic structure which accepts pollen and facilitates fertilization. Floral development is mostly governed by the MIKC‐MADS box transcription factors (Coen & Meyerowitz, 1991). There are 43 known MIKC MADS genes in the maize genome (Zhao et al., 2010) and 16 of them were found to be down‐regulated in stressed ears, which is about 40% of all maize MADS box genes (Table 4). MADS proteins work in quadruple complexes and their combination defines specification of the floral organs (Coen & Meyerowitz, 1991). To some extent this model is pertinent for flower morphogenesis in grasses as well (Ciaffi, Paolacci, Tanzarella, & Porceddu, 2011). Stress sensitive MADS genes represent all functional A, B, C, D, and E classes (Table 4). MADS box genes that encode proteins that form the heterodimers ZAG1 and ZAG3 (BDE1) (Thompson et al., 2009) were both down‐regulated under stress. Interestingly, *ZMM16* which was down‐regulated by stress in our study (Table 4) was identified as a drought QTL in tropical maize suggesting a putative role in stress adaptation (Almeida et al., 2014). In addition, *ZMM16* was also identified as a potential QTL candidate for accumulation of the ABA metabolite phaseic acid in ears (Setter et al., 2011). Non‐MADS floral genes APETALA2‐like transcription factors (*TS6, tasselseed6*) and *DL1* (*drooping leaves*), homologs of the rice YABBY transcription factor, were also down‐regulated in stressed ears.

The key gene for embryo sac development, *IG1* (*indeterminate gametophyte*) (Evans, 2007), was also down‐regulated by stress. This finding sheds light on the observation that even short periods of water limitation can lead to abnormalities in embryo sac development (Moss & Downey, 1971). The down‐regulation of the *FERONIA* homolog is intriguing. In Arabidopsis a receptor‐like kinase FERONIA is required for the pollen tube interaction with the synergids (Escobar‐Restrepo et al., 2007). The function of the maize homolog has yet to be shown, but we can speculate that down‐regulation of the *FERONIA* homolog might impair efficacy of fertilization under abiotic stress conditions. Collectively, many genes involved in gynoecium development are suppressed under drought stress, which ultimately leads to the formation of defective ovaries and a failure to produce kernels. Due to acropetal ear development, this process is most prominent at the ear tip where the youngest florets are positioned.

## 5 CONCLUSION

The monoecious nature of maize allowed us to test and identify differential effects of abiotic stress on three key plant organs: leaf, ear, and tassel. Our results indicate that each organ perceives different levels of stress which in turn drive a differential growth and developmental response. The tassel displayed the lowest transcriptional response to stress relative to the other organs and thus seems to be relatively well insulated from abiotic stress. This may be related to the evolutionary strategy prioritizing pollen which can be dispersed over wide distance over female organ development, which are immobilized in the drought‐stressed environment. In contrast the ear has distinct and independent responses to drought in that organogenesis (spikelet initiation) appeared to be relatively stable under drought stress while organ extension (ear length) was significantly altered. The reduction in ear growth under drought stress was associated with the down‐regulation of gene expression from pathways involved in DNA replication, cell‐cycle, and cell division. This is consistent with published observations that inhibition of cell division is a common response to drought stress in plant organs such as silks (Fuad‐Hassan et al., 2008), ovaries (Kakumanu et al., 2012), endosperm (Setter & Flannigan, 2001), and leaf meristem (Avramova et al., 2015). Our results also suggest that jasmonates may be mediators of cell cycle suppression (Kazan, 2015; Pauwels et al., 2008), but this hypothesis would need to be tested.

It is important to emphasize that the B73 line used in this study was released in 1972 and represents “older genetics” that is more susceptible to abiotic stress. In recent years advanced drought tolerant germplasm has been created; for example, Optimum^®^ AQUAmax^™^ hybrids (Gaffney et al., 2015). It would be interesting to contrast the phenotypic and transcriptional changes in the parents of these modern drought tolerant hybrids with that of B73 to determine which attributes have been altered by breeding for abiotic stress tolerance.

## CONFLICT OF INTEREST

The authors declare no conflict of interest associated with the work described in this manuscript.

## AUTHORS’ CONTRIBUTIONS

O.D. conceived and designed research. G.X.Y. and S.T. analyzed data. X.M., J.X., S.E. L.S. S.C., and G.Z.H. performed research. O.D. and S.T. wrote the article.

## References

[pld3129-bib-0001] Abendroth, L. J. , Elmore, M. L. , Boyer, M. J. , & Marlay, S. K. (2011). Corn growth and development. Ames, IA: Iowa Iowa State University.

[pld3129-bib-0002] Almeida, G. D. , Nair, S. , Borem, A. , Cairns, J. , Trachsel, S. , Ribaut, J. M. , … Babu, R. (2014). Molecular mapping across three populations reveals a QTL hotspot region on chromosome 3 for secondary traits associated with drought tolerance in tropical maize. Molecular Breeding : New Strategies in Plant Improvement, 34, 701–715. 10.1007/s11032-014-0068-5 25076840 PMC4092235

[pld3129-bib-0003] Al‐Whaibi, M. H. (2011). Plant heat‐shock proteins: A mini review. Journal of King Saud University Science, 23, 139–150. 10.1016/j.jksus.2010.06.022

[pld3129-bib-0004] Araus, J. L. , Serret, M. D. , & Edmeades, G. O. (2012). Phenotyping maize for adaptation to drought. Frontiers in Physiology, 3, 305 10.3389/fphys.2012.00305 22934056 PMC3429076

[pld3129-bib-0005] Avramova, V. , AbdElgawad, H. , Zhang, Z. , Fotschki, B. , Casadevall, R. , Vergauwen, L. , … Beemster, G. T. (2015). Drought induces distinct growth response, protection, and recovery mechanisms in the maize leaf growth zone. Plant Physiology, 169, 1382–1396. 10.1104/pp.15.00276 26297138 PMC4587441

[pld3129-bib-0006] Badicean, D. , Scholten, S. , & Jacota, A. (2011). Transcriptional profiling of Zea mays genotypes with different drought tolerances–New perspectives for gene expression markers selection. Maydica, 56, 61–69.

[pld3129-bib-0007] Barazesh, S. , & McSteen, P. (2008). Hormonal control of grass inflorescence development. Trends in Plant Science, 13, 656–662. 10.1016/j.tplants.2008.09.007 18986827

[pld3129-bib-0008] Barazesh, S. , Nowbakht, C. , & McSteen, P. (2009). sparse inflorescence1, barren inflorescence1 and barren stalk1 promote cell elongation in maize inflorescence development. Genetics, 182, 403–406. 10.1534/genetics.108.099390 19279326 PMC2674837

[pld3129-bib-0009] Barker, T. , Campos, H. , Cooper, M. , Dolan, D. , Edmeades, G. , Habben, J. , … Zinselmeier, C. (2005). Improving drought tolerance in maize In Janick J. (Ed.), Plant breeding reviews (pp. 173–253). New York, NY: John Wiley & Sons Inc.

[pld3129-bib-0010] Benjamini, Y. , Drai, D. , Elmer, G. , Kafkafi, N. , & Golani, I. (2001). Controlling the false discovery rate in behavior genetics research. Behavioural Brain Research, 125, 279–284. 10.1016/S0166-4328(01)00297-2 11682119

[pld3129-bib-0011] Blum, A. (2014). Genomics for drought resistance – getting down to earth. Functional Plant Biology, 41, 1191 10.1071/FP14018 32481068

[pld3129-bib-0012] Boyer, J. S. , Byrne, P. , Cassman, K. G. , Cooper, M. , Delmer, D. , Greene, T. , … Gaffney, J. (2013). The U.S. drought of 2012 in perspective: A call to action. Global Food Security, 2, 139–143. 10.1016/j.gfs.2013.08.002

[pld3129-bib-0013] Boyer, J. S. , & Westgate, M. E. (2004). Grain yields with limited water. Journal of Experimental Botany, 55, 2385–2394. 10.1093/jxb/erh219 15286147

[pld3129-bib-0014] Campos, H. , Cooper, M. , Edmeades, G. O. , Löffler, C. , Schussler, J. R. , & Ibañez, M. (2006). Changes in drought tolerance in maize associated with fifty years of breeding for yield in the U.S. corn belt. Maydica, 51, 369–381.

[pld3129-bib-0015] Campos, H. , Cooper, M. , Habben, J. E. , Edmeades, G. O. , & Schussler, J. R. (2004). Improving drought tolerance in maize: A view from industry. Field Crops Research, 90, 19–34. 10.1016/j.fcr.2004.07.003

[pld3129-bib-5000] Cheng, P. C. , Greyson, R. I. , & Walden, D. B. (1983). Organ initiation and the development of unisexual flowers in the tassel and ear of Zea mays. American Journal of Botany, 70(3), 450–462.

[pld3129-bib-0016] Chuck, G. , Muszynski, M. , Kellogg, E. , Hake, S. , & Schmidt, R. J. (2002). The control of spikelet meristem identity by the branched silkless1 gene in maize. Science, 298, 1238–1241. 10.1126/science.1076920 12424380

[pld3129-bib-0017] Ciaffi, M. , Paolacci, A. R. , Tanzarella, O. A. , & Porceddu, E. (2011). Molecular aspects of flower development in grasses. Sexual Plant Reproduction, 24, 247–282. 10.1007/s00497-011-0175-y 21877128

[pld3129-bib-0018] Coen, E. S. , & Meyerowitz, E. M. (1991). The war of the whorls: Genetic interactions controlling flower development. Nature, 353, 31–37. 10.1038/353031a0 1715520

[pld3129-bib-0019] Cooper, M. , Gho, C. , Leafgren, R. , Tang, T. , & Messina, C. (2014). Breeding drought‐tolerant maize hybrids for the US corn‐belt: Discovery to product. Journal of Experimental Botany, 65, 6191–6204. 10.1093/jxb/eru064 24596174

[pld3129-bib-0020] Deikman, J. , Petracek, M. , & Heard, J. E. (2012). Drought tolerance through biotechnology: Improving translation from the laboratory to farmers’ fields. Current Opinion in Biotechnology, 23, 243–250. 10.1016/j.copbio.2011.11.003 22154468

[pld3129-bib-0021] Escobar‐Restrepo, J. M. , Huck, N. , Kessler, S. , Gagliardini, V. , Gheyselinck, J. , Yang, W. C. , & Grossniklaus, U. (2007). The FERONIA receptor‐like kinase mediates male‐female interactions during pollen tube reception. Science, 317, 656–660. 10.1126/science.1143562 17673660

[pld3129-bib-0022] Evans, M. M. (2007). The indeterminate gametophyte1 gene of maize encodes a LOB domain protein required for embryo Sac and leaf development. Plant Cell, 19, 46–62. 10.1105/tpc.106.047506 17209126 PMC1820972

[pld3129-bib-0023] Fernandes, J. , Morrow, D. J. , Casati, P. , & Walbot, V. (2008). Distinctive transcriptome responses to adverse environmental conditions in Zea mays L. Plant Biotechnology Journal, 6, 782–798. 10.1111/j.1467-7652.2008.00360.x 18643947

[pld3129-bib-0024] Fernandez, D. S. , & Castrillo, M. (1999). Leaf rolling initiation. Photosynthetica, 37, 493–497. 10.1023/A:1007124214141

[pld3129-bib-0025] Fuad‐Hassan, A. , Tardieu, F. , & Turc, O. (2008). Drought‐induced changes in anthesis‐silking interval are related to silk expansion: A spatio‐temporal growth analysis in maize plants subjected to soil water deficit. Plant, Cell & Environment, 31, 1349–1360. 10.1111/j.1365-3040.2008.01839.x 18518916

[pld3129-bib-0026] Gaffney, J. , Schussler, J. , Löffler, C. , Cai, W. , Paszkiewicz, S. , Messina, C. , … Cooper, M. (2015). Industry‐scale evaluation of maize hybrids selected for increased yield in drought‐stress conditions of the US corn belt. Crop Science, 55, 1608–1618. 10.2135/cropsci2014.09.0654

[pld3129-bib-0027] Gallavotti, A. (2013). The role of auxin in shaping shoot architecture. Journal of Experimental Botany, 64, 2593–2608. 10.1093/jxb/ert141 23709672

[pld3129-bib-0028] Gallavotti, A. , Barazesh, S. , Malcomber, S. , Hall, D. , Jackson, D. , Schmidt, R. J. , & McSteen, P. (2008). Sparse inflorescence1 encodes a monocot‐specific YUCCA‐like gene required for vegetative and reproductive development in maize. Proceedings of the National Academy of Sciences of the United States of America, 105, 15196–15201. 10.1073/pnas.0805596105 18799737 PMC2567514

[pld3129-bib-0029] Gallavotti, A. , Malcomber, S. , Gaines, C. , Stanfield, S. , Whipple, C. , Kellogg, E. , & Schmidt, R. J. (2011). BARREN STALK FASTIGIATE1 is an AT‐hook protein required for the formation of maize ears. Plant Cell, 23, 1756–1771. 10.1105/tpc.111.084590 21540434 PMC3123940

[pld3129-bib-0030] Gallavotti, A. , Yang, Y. , Schmidt, R. J. , & Jackson, D. (2008). The relationship between auxin transport and maize branching. Plant Physiology, 147, 1913–1923. 10.1104/pp.108.121541 18550681 PMC2492655

[pld3129-bib-0031] Gallavotti, A. , Zhao, Q. , Kyozuka, J. , Meeley, R. B. , Ritter, M. K. , Doebley, J. F. , … Schmidt, R. J. (2004). The role of barren stalk1 in the architecture of maize. Nature, 432, 630–635. 10.1038/nature03148 15577912

[pld3129-bib-0032] Habben, J. E. , Bao, X. , Bate, N. J. , DeBruin, J. L. , Dolan, D. , Hasegawa, D. , … Schussler, J. R. (2014). Transgenic alteration of ethylene biosynthesis increases grain yield in maize under field drought‐stress conditions. Plant Biotechnology Journal, 12, 685–693. 10.1111/pbi.12172 24618117

[pld3129-bib-0033] Haruta, M. , Sabat, G. , Stecker, K. , Minkoff, B. B. , & Sussman, M. R. (2014). A peptide hormone and its receptor protein kinase regulate plant cell expansion. Science, 343, 408–411. 10.1126/science.1244454 24458638 PMC4672726

[pld3129-bib-0034] Hauser, F. , Waadt, R. , & Schroeder, J. I. (2011). Evolution of abscisic acid synthesis and signaling mechanisms. Current Biology, 21, R346–R355. 10.1016/j.cub.2011.03.015 21549957 PMC3119208

[pld3129-bib-0035] Herrero, M. P. , & Johnson, R. R. (1980a). Drought stress and its effects on maize reproductive systems. Crop Science, 21, 105–110.

[pld3129-bib-0036] Herrero, M. P. , & Johnson, R. R. (1980b). High temperature stress and pollen viability of maize. Crop Science, 20, 796–800. 10.2135/cropsci1980.0011183X002000060030x

[pld3129-bib-0037] Hu, X. , Yang, Y. , Gong, F. , Zhang, D. , Zhang, L. , Wu, L. , … Wang, W. (2015). Protein sHSP26 improves chloroplast performance under heat stress by interacting with specific chloroplast proteins in maize (Zea mays). Journal of Proteomics, 115, 81–92. 10.1016/j.jprot.2014.12.009 25540934

[pld3129-bib-0038] Johnson, S. M. , Lim, F. L. , Finkler, A. , Fromm, H. , Slabas, A. R. , & Knight, M. R. (2014). Transcriptomic analysis of Sorghum bicolor responding to combined heat and drought stress. BMC Genomics, 15, 456 10.1186/1471-2164-15-456 24916767 PMC4070570

[pld3129-bib-0039] Kakumanu, A. , Ambavaram, M. M. , Klumas, C. , Krishnan, A. , Batlang, U. , Myers, E. , … Pereira, A. (2012). Effects of drought on gene expression in maize reproductive and leaf meristem tissue revealed by RNA‐Seq. Plant Physiology, 160, 846–867. 10.1104/pp.112.200444 22837360 PMC3461560

[pld3129-bib-0040] Kazan, K. (2015). Diverse roles of jasmonates and ethylene in abiotic stress tolerance. Trends in Plant Science, 20, 219–229. 10.1016/j.tplants.2015.02.001 25731753

[pld3129-bib-0041] Koops, P. , Pelser, S. , Ignatz, M. , Klose, C. , Marrocco‐Selden, K. , & Kretsch, T. (2011). EDL3 is an F‐box protein involved in the regulation of abscisic acid signalling in Arabidopsis thaliana. Journal of Experimental Botany, 62, 5547–5560. 10.1093/jxb/err236 21831845 PMC3223051

[pld3129-bib-0042] Li, H. Y. , Wang, T. Y. , Shi, Y. S. , Fu, J. J. , Song, Y. C. , Wang, G. Y. , & Li, Y. (2007). Isolation and characterization of induced genes under drought stress at the flowering stage in maize (Zea mays). DNA Sequence : The Journal of DNA Sequencing and Mapping, 18, 445–460. 10.1080/10425170701292051 17676474

[pld3129-bib-0043] Liu, Z. , Zhang, S. , Sun, N. , Liu, H. , Zhao, Y. , Liang, Y. , … Han, Y. (2015). Functional diversity of jasmonates in rice. Rice, 8, 5 10.1186/s12284-015-0042-9 PMC4773313 26054241

[pld3129-bib-0044] Luo, M. , Liu, J. , Lee, R. D. , Scully, B. T. , & Guo, B. (2010). Monitoring the expression of maize genes in developing kernels under drought stress using oligo‐microarray. Journal of Integrative Plant Biology, 52, 1059–1074. 10.1111/j.1744-7909.2010.01000.x 21106005

[pld3129-bib-0045] Mallya, G. , Zhao, L. , Song, X. , Niyogi, D. , & Govindaraju, R. (2013). 2012 midwest drought in the United States. Journal of Hydrologic Engineering, 18, 737–745. 10.1061/(ASCE)HE.1943-5584.0000786

[pld3129-bib-0046] Marino, R. , Ponnaiah, M. , Krajewski, P. , Frova, C. , Gianfranceschi, L. , Pe, M. E. , & Sari‐Gorla, M. (2009). Addressing drought tolerance in maize by transcriptional profiling and mapping. Molecular Genetics and Genomics, 281, 163–179. 10.1007/s00438-008-0401-y 19018570

[pld3129-bib-0047] McSteen, P. , Malcomber, S. , Skirpan, A. , Lunde, C. , Wu, X. , Kellogg, E. , & Hake, S. (2007). barren inflorescence2 Encodes a co‐ortholog of the PINOID serine/threonine kinase and is required for organogenesis during inflorescence and vegetative development in maize. Plant Physiology, 144, 1000–1011. 10.1104/pp.107.098558 17449648 PMC1914211

[pld3129-bib-0048] Mittler, R. , & Blumwald, E. (2010). Genetic engineering for modern agriculture: Challenges and perspectives. Annual Review of Plant Biology, 61, 443–462. 10.1146/annurev-arplant-042809-112116 20192746

[pld3129-bib-0049] Moss, G. I. , & Downey, L. A. (1971). Influence of drought stress on female gametophyte development in corn (Zea mays L.) and subsequent grain yield. Crop Science, 11, 368–372. 10.2135/cropsci1971.0011183X001100030017x

[pld3129-bib-0050] Pautler, M. , Eveland, A. L. , LaRue, T. , Yang, F. , Weeks, R. , Lunde, C. , … Jackson, D. (2015). FASCIATED EAR4 encodes a bZIP transcription factor that regulates shoot meristem size in maize. Plant Cell, 27, 104–120. 10.1105/tpc.114.132506 25616871 PMC4330574

[pld3129-bib-0051] Pauwels, L. , Inze, D. , & Goossens, A. (2009). Jasmonate‐inducible gene: What does it mean? Trends in Plant Science, 14, 87–91. 10.1016/j.tplants.2008.11.005 19162528

[pld3129-bib-0052] Pauwels, L. , Morreel, K. , De Witte, E. , Lammertyn, F. , Van Montagu, M. , Boerjan, W. , … Goossens, A. (2008). Mapping methyl jasmonate‐mediated transcriptional reprogramming of metabolism and cell cycle progression in cultured Arabidopsis cells. Proceedings of the National Academy of Sciences of the United States of America, 105, 1380–1385. 10.1073/pnas.0711203105 18216250 PMC2234147

[pld3129-bib-0054] Phillips, K. A. , Skirpan, A. L. , Liu, X. , Christensen, A. , Slewinski, T. L. , Hudson, C. , … McSteen, P. (2011). vanishing tassel2 encodes a grass‐specific tryptophan aminotransferase required for vegetative and reproductive development in maize. The Plant Cell, 23, 550–566. 10.1105/tpc.110.075267 21335375 PMC3077783

[pld3129-bib-0055] Savchenko, T. , Kolla, V. A. , Wang, C. Q. , Nasafi, Z. , Hicks, D. R. , Phadungchob, B. , … Dehesh, K. (2014). Functional convergence of oxylipin and abscisic acid pathways controls stomatal closure in response to drought. Plant Physiology, 164, 1151–1160. 10.1104/pp.113.234310 24429214 PMC3938610

[pld3129-bib-0056] Savchenko, T. V. , Zastrijnaja, O. M. , & Klimov, V. V. (2014). Oxylipins and plant abiotic stress resistance. Biochemistry, 79, 362–375. 24910209 10.1134/S0006297914040051

[pld3129-bib-0057] Schoper, J. B. , Lambert, R. J. , & Vasilas, B. L. (1985). Maize pollen viability and ear receptivity under water and high temperature stress. Crop Science, 26(5), 1029–1033.

[pld3129-bib-0058] Schussler, J. R. , & Westgate, M. E. (1990). Maize kernel set at low water potential: II. Sensitivity to reduced assimilates at pollination. Crop Science, 31, 1196–1203. 10.2135/cropsci1991.0011183x003100050024x

[pld3129-bib-0059] Setter, T. L. , & Flannigan, B. A. (2001). Water deficit inhibits cell division and expression of transcripts involved in cell proliferation and endoreduplication in maize endosperm. Journal of Experimental Botany, 52, 1401–1408. 10.1093/jexbot/52.360.1401 11457899

[pld3129-bib-0060] Setter, T. L. , Yan, J. , Warburton, M. , Ribaut, J. M. , Xu, Y. , Sawkins, M. , … Gore, M. A. (2011). Genetic association mapping identifies single nucleotide polymorphisms in genes that affect abscisic acid levels in maize floral tissues during drought. Journal of Experimental Botany, 62, 701–716. 10.1093/jxb/erq308 21084430 PMC3003815

[pld3129-bib-0061] Shan, X. , Li, Y. , Jiang, Y. , Jiang, Z. , Hao, W. , & Yuan, Y. (2013). Transcriptome profile analysis of maize seedlings in response to high‐salinity, drought and cold stresses by deep sequencing. Plant Molecular Biology Reporter, 31, 1485–1491. 10.1007/s11105-013-0622-z

[pld3129-bib-0062] Shaw, R. H. (1977). Water use and requirements of maize‐a review (pp. 119–134). In Agrometeorology of the Maize (corn) Crop. Publication 480. Geneva, Switzerland: World Meteorological Organization.

[pld3129-bib-0063] Shi, J. , Habben, J. E. , Archibald, R. L. , Drummond, B. J. , Chamberlin, M. A. , Williams, R. W. , … Weers, B. P. (2015). Overexpression of ARGOS genes modifies plant sensitivity to ethylene, leading to improved drought tolerance in both arabidopsis and maize. Plant Physiology, 169, 266–282. 10.1104/pp.15.00780 26220950 PMC4577415

[pld3129-bib-0064] Shyu, C. , & Brutnell, T. P. (2015). Growth‐defence balance in grass biomass production: The role of jasmonates. Journal of Experimental Botany, 66(14), 4165–4176. 10.1093/jxb/erv011 25711704

[pld3129-bib-0065] Tanaka, W. , Pautler, M. , Jackson, D. , & Hirano, H. Y. (2013). Grass meristems II: Inflorescence architecture, flower development and meristem fate. Plant & Cell Physiology, 54, 313–324. 10.1093/pcp/pct016 23378448

[pld3129-bib-0066] Thatcher, S. R. , Danilevskaya, O. N. , Meng, X. , Beatty, M. , Zastrow‐Hayes, G. , Harris, C. , … Li, B. (2016). Genome‐wide analysis of alternative splicing during development and drought stress in Zea mays. Plant Physiology, 170, 586–599. 10.1104/pp.15.01267 26582726 PMC4704579

[pld3129-bib-0067] Thatcher, S. R. , Zhou, W. , Leonard, A. , Wang, B. B. , Beatty, M. , Zastrow‐Hayes, G. , … Li, B. (2014). Genome‐wide analysis of alternative splicing in Zea mays: Landscape and genetic regulation. Plant Cell, 26, 3472–3487. 10.1105/tpc.114.130773 25248552 PMC4213170

[pld3129-bib-0068] Thompson, B. E. , Bartling, L. , Whipple, C. , Hall, D. H. , Sakai, H. , Schmidt, R. , & Hake, S. (2009). bearded‐ear Encodes a MADS box transcription factor critical for maize floral development. Plant Cell, 21, 2578–2590. 10.1105/tpc.109.067751 19749152 PMC2768933

[pld3129-bib-0069] Wasternack, C. , & Hause, B. (2013). Jasmonates: Biosynthesis, perception, signal transduction and action in plant stress response, growth and development. An update to the 2007 review in Annals of Botany. Annals of Botany, 111, 1021–1058. 10.1093/aob/mct067 23558912 PMC3662512

[pld3129-bib-0070] Westgate, M. E. , & Grant, D. L. (1989). Water deficits and reproduction in maize : Response of the reproductive tissue to water deficits at anthesis and mid‐grain fill. Plant Physiology, 91, 862–867. 10.1104/pp.91.3.862 16667149 PMC1062088

[pld3129-bib-0071] Yang, S. , Logan, J. , & Coffey, D. L. (1995). Mathematical formulae for calculating the base temperature for growing degree days. Agricultural and Forest Meteorology, 74, 61–74. 10.1016/0168-1923(94)02185-M

[pld3129-bib-0072] Yue, G. , Zhuang, Y. , Li, Z. , Sun, L. , & Zhang, J. (2008). Differential gene expression analysis of maize leaf at heading stage in response to water‐deficit stress. Bioscience Reports, 28, 125–134. 10.1042/BSR20070023 18422487

[pld3129-bib-0073] Zhao, Y. , Li, X. , Chen, W. , Peng, X. , Cheng, X. , Zhu, S. , & Cheng, B. (2010). Whole‐genome survey and characterization of MADS‐box gene family in maize and sorghum. Plant Cell, Tissue and Organ Culture, 105, 159–173.

[pld3129-bib-0074] Zheng, J. , Fu, J. , Gou, M. , Huai, J. , Liu, Y. , Jian, M. , … Wang, G. (2010). Genome‐wide transcriptome analysis of two maize inbred lines under drought stress. Plant Molecular Biology, 72, 407–421. 10.1007/s11103-009-9579-6 19953304

[pld3129-bib-0075] Zhuang, Y. , Ren, G. , Yue, G. , Li, Z. , Qu, X. , Hou, G. , … Zhang, J. (2007). Effects of water‐deficit stress on the transcriptomes of developing immature ear and tassel in maize. Plant Cell Reports, 26, 2137–2147. 10.1007/s00299-007-0419-3 17668218

[pld3129-bib-0076] Zinselmeier, C. , Sun, Y. , Helentjaris, T. , Beatty, M. , Yang, S. , Smith, H. , & Habben, J. (2002). The use of gene expression profiling to dissect the stress sensitivity of reproductive development in maize. Field Crops Research, 75, 111–121. 10.1016/S0378-4290(02)00021-7

