# Supplementary material for: Developmental and transcriptional responses of maize to drought stress under field conditions
Source: Plant Direct. 2019 May 7;3(5):e00129. doi: 10.1002/pld3.129 (PMC6589525; doi:10.1002/pld3.129)
Supplement: Supplementary file 1 [file PLD3-3-e00129-s001.pdf]

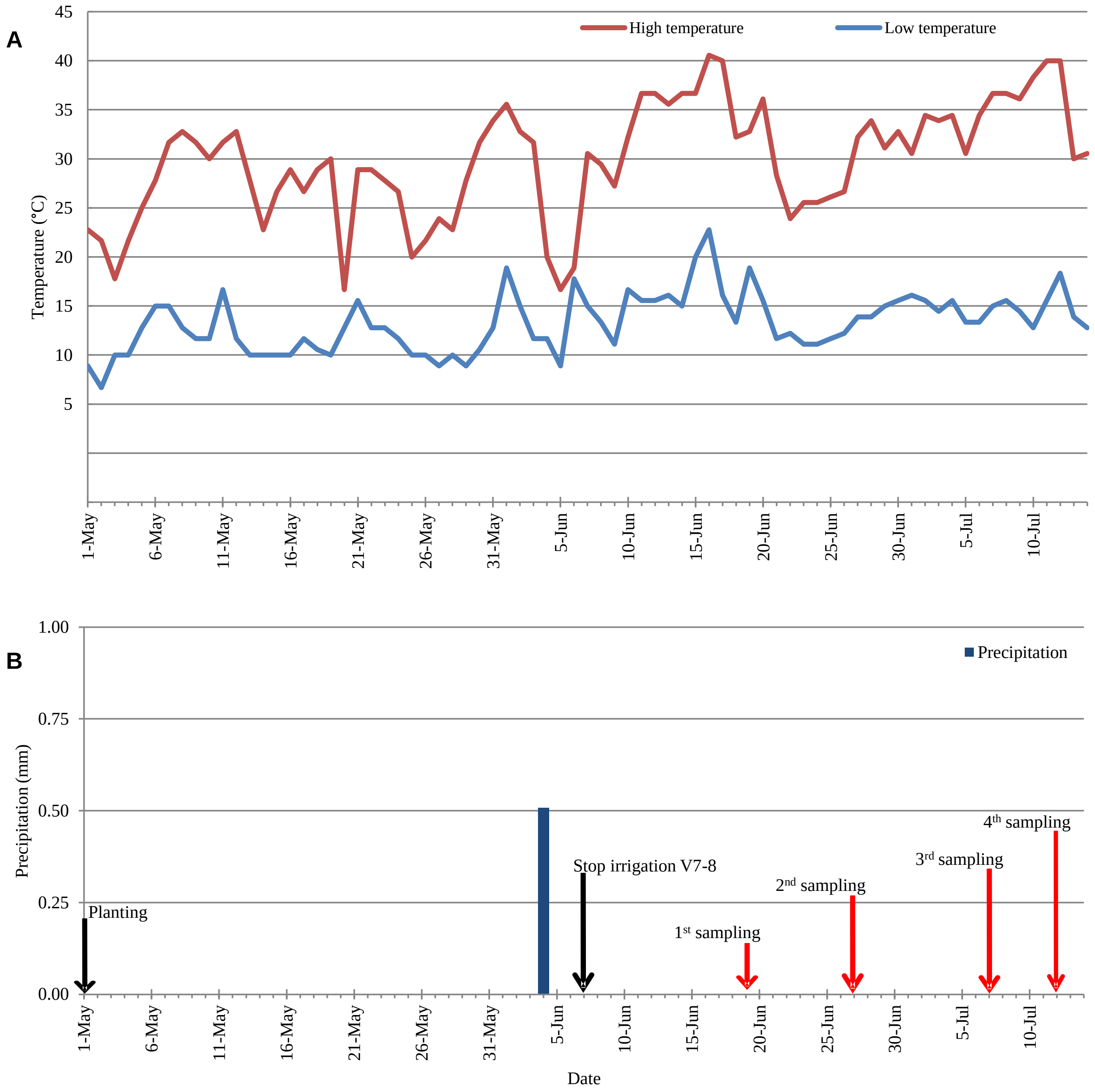

Supplemental Figure S1. 2012 weather and sampling schedule at Woodland, CA (38° 40' N, 121° 50' W). Daily temperature (^{0}C) patterns during the course of the experiment (A). Plots were planted (B) on May 1st, 2012 and fully irrigated until June 7th; thereafter the drought plots were never re-watered, while well-watered plots continued to be irrigated. On June 7th plants had reached the V7-V8 stages and ears were just initiated in the leaf axil having 2-4 spikelet pair meristems. The first tissue sampling was done on June 19th after 11 days of drought, when plants reached the V11-12 stages. The second tissue sampling was done on June 27th after 18 days of drought, when plants reached the V13-14 stages. The third tissue sampling was done on July 7th after 27 days of drought, when plants reached the V17-18 stages. The fourth tissue sampling was done on July 12th after 32 days of drought, when plants were at the silking R1 stage in the well-watered plots. No exerted silks were observed in the drought plots.

A

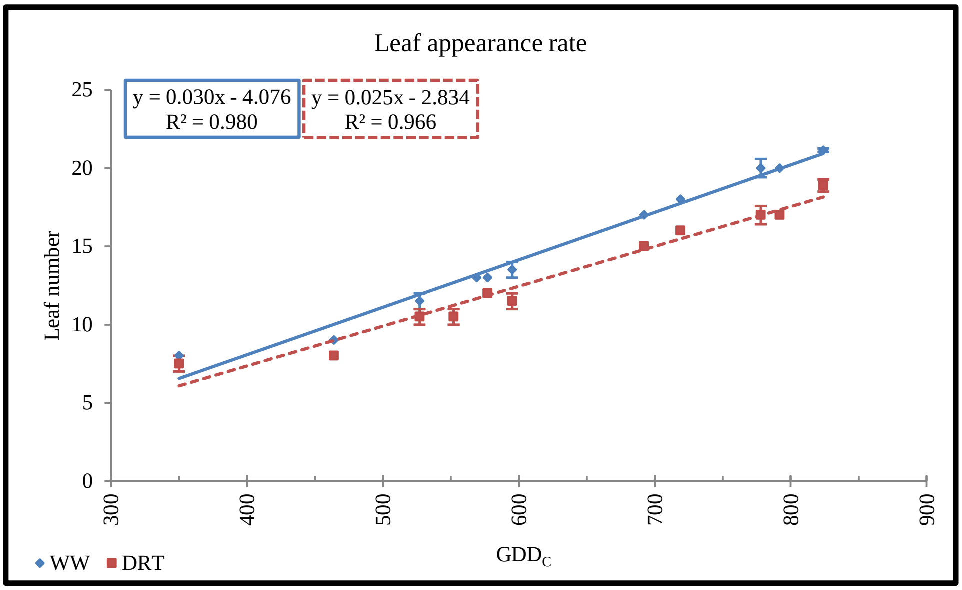

B

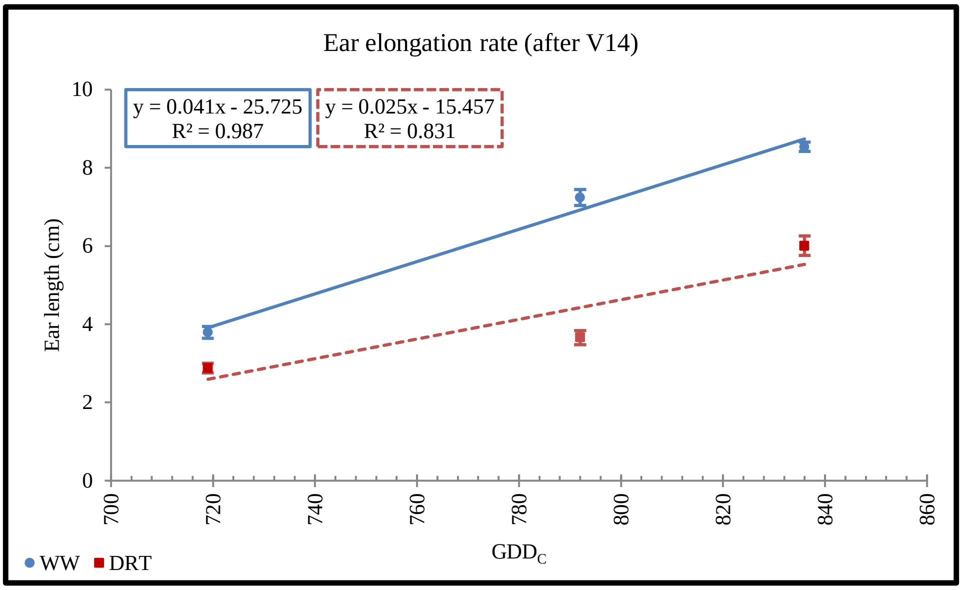

Supplemental Figure S2. Linear regression models of leaf appearance rate (A) and ear elongation rate (B) in the field experiment Woodland, CA, 2012, under well watered (WW) (blue diamond) and drought (DRT) (red square) treatments. Error bars denote standard error. GDD_C, growing degree days in celsius. Linear regression formula with R^2 value. Data are averaged by day and treatment.

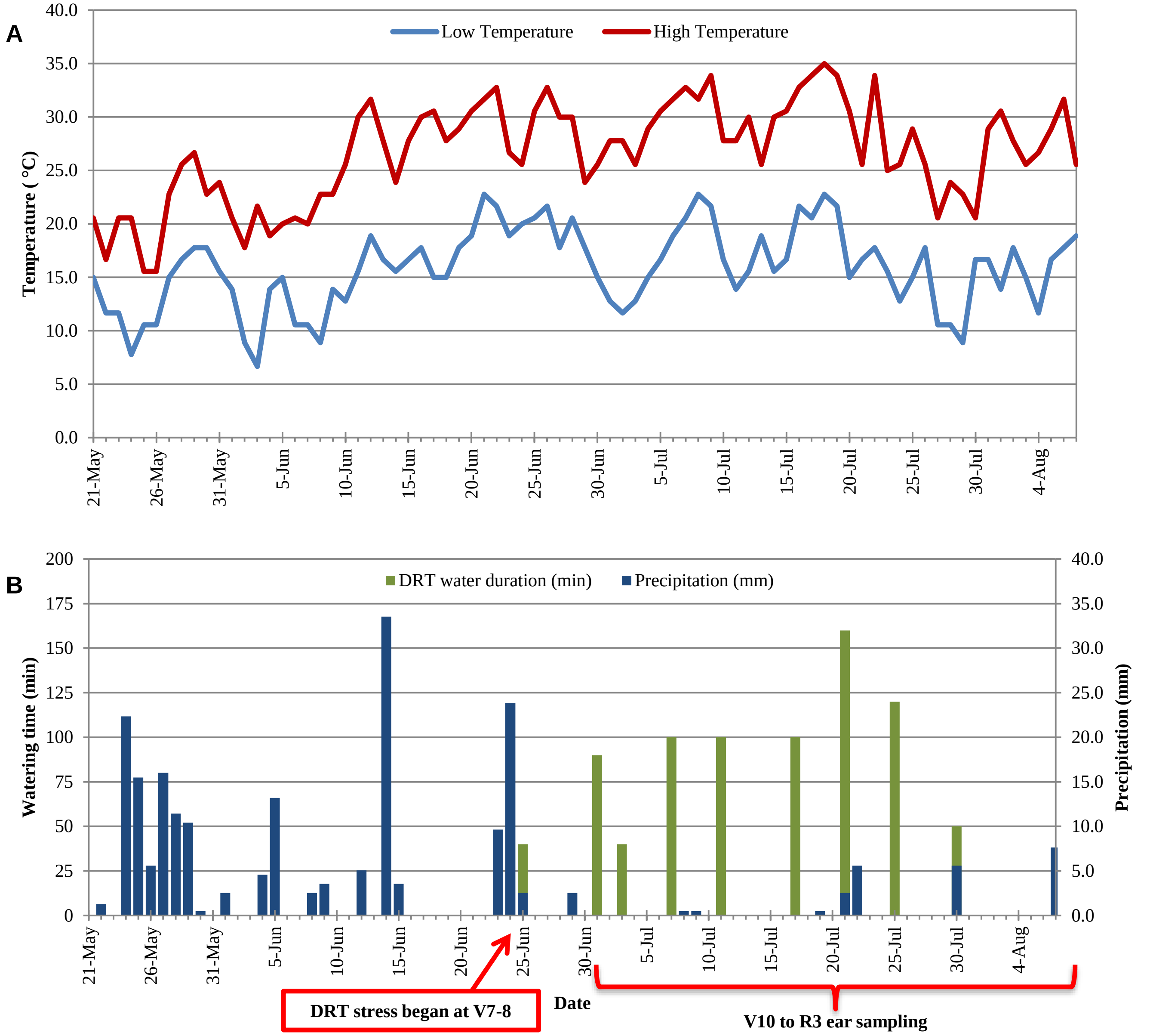

Supplemental Figure S3. 2013 weather and irrigation schedule at Johnston, IA (41° 40' N, 93° 42' W). Daily temperature (^{0}C) patterns during the course of the experiment (A). Pots were planted (B) on May 22nd 2013 and full irrigated until June 25th when plants reached V7-V8 stages. Then a limited water regime was applied. The pots designated as drought treatment were not irrigated until 50% of the plants showed obvious leaf wilting. Once this occurred, pots were re-watered to full soil capacity and the dry-down cycle was repeated 8 times from the stage V8 to R3 in the course of the experiment. This water regime simulated drought stress at flowering time.

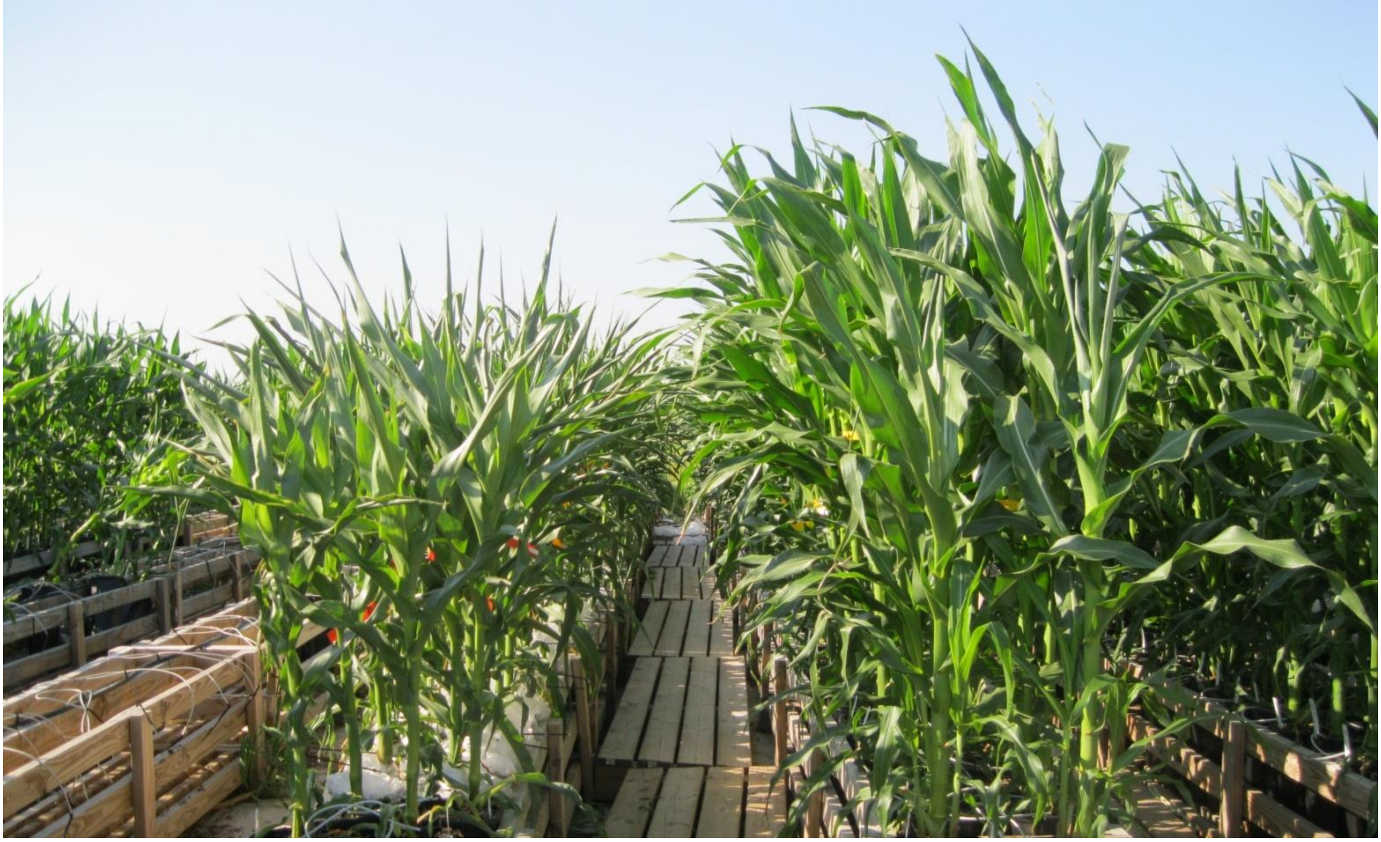

Supplemental Figure S4. Overview of the field pot study at Johnston IA, 2013**.** Drought (left) and well-watered (right) plants at the V14 stage.

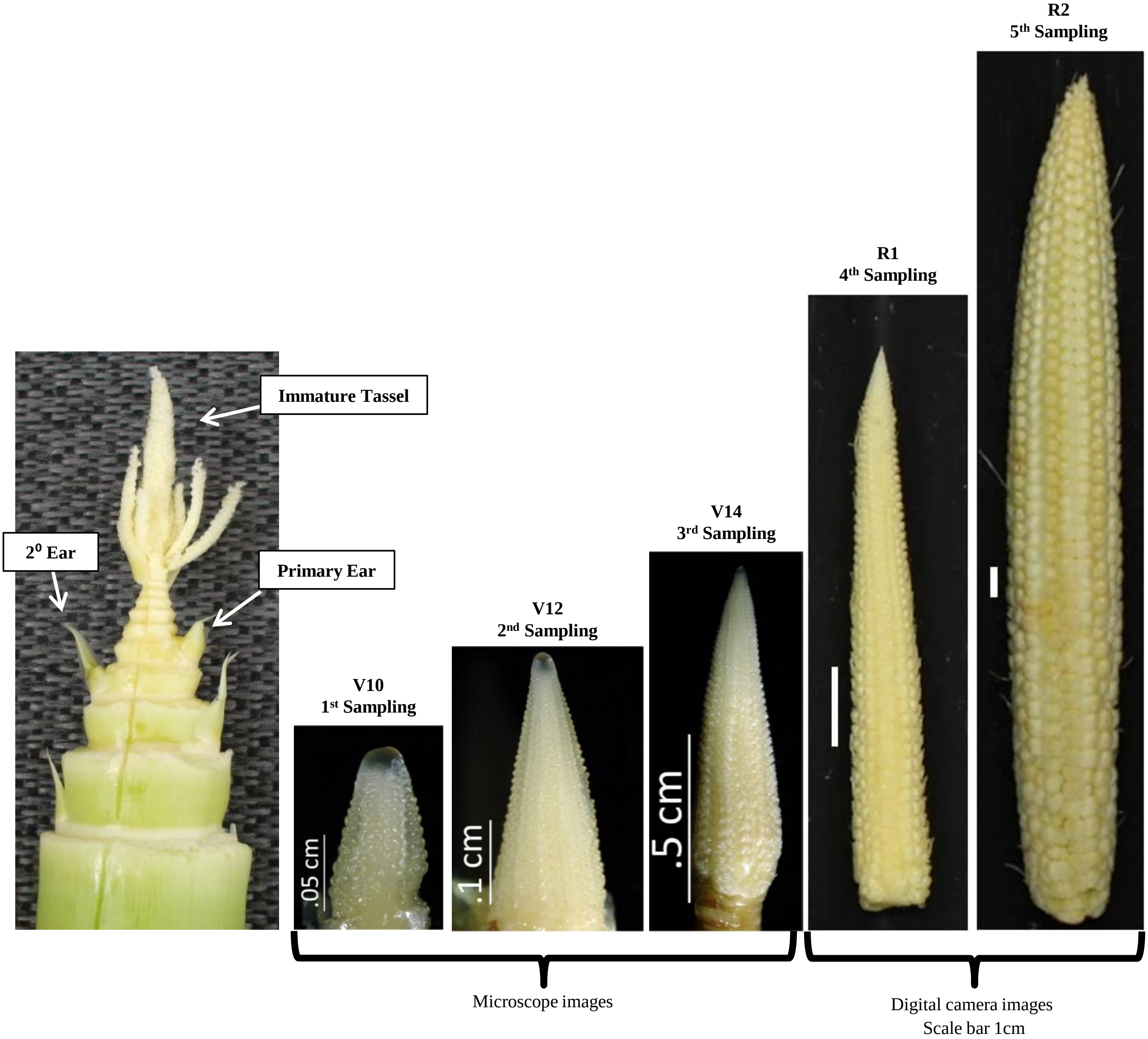

Supplemental Figure S5. Representative images of the primary ear under well-watered conditions in the field pot study. At V10, ears were at early development having the translucent inflorescence meristem (IM) and the spikelet pair meristem (SPM). During V12-V14, ears continued to have the translucent IM initiating more SPMs and shoot meristems. After V14, the IM was consumed and no more SPMs were initiated. At every stage, we manually counted the spikelet number per two opposite rows on the dissected ears and took notes on the IM translucency to record the termination of ear development.

A

B

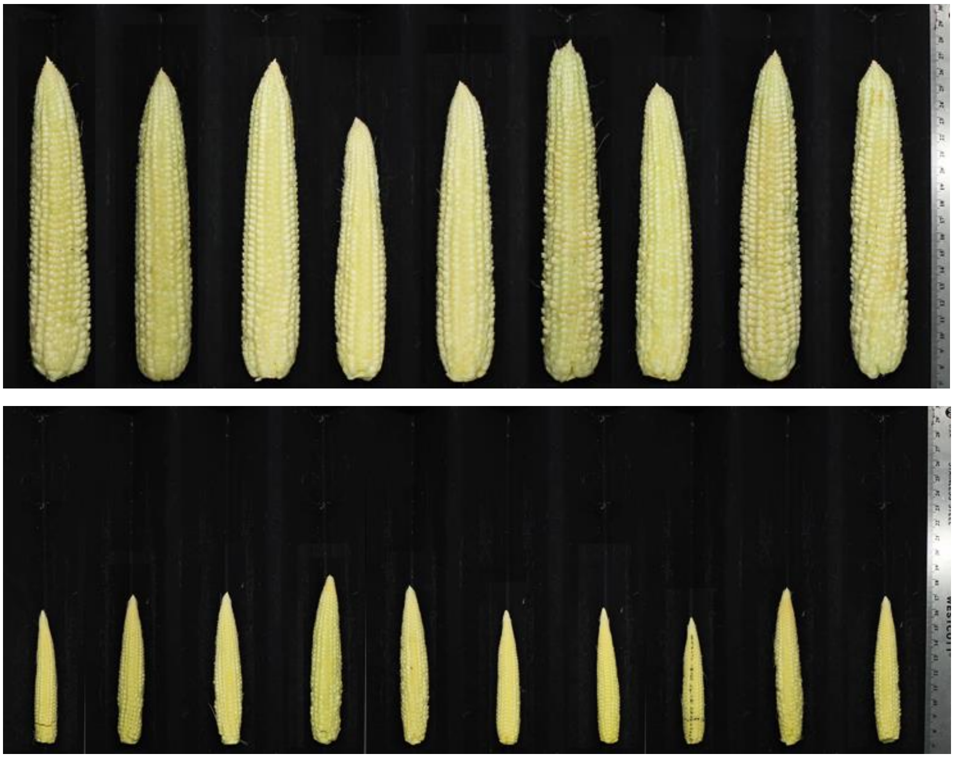

Supplemental Figure S6. Representative ear images of B73 plants from the field pot study. Well-watered (A) and drought (B) ears at the R2 stage (922 growing degree days)

Tissue	No. Transcripts WW	No. Transcripts DRT	DE genes
Leaf	31862	32987	3454
Ear	34145	32794	6946
Tassel	37030	37516	19850

Supplemental Figure S7. Transcript abundance and differentially expressed (DE) genes across all sampling times in vegetative and reproductive tissues, Woodland, CA under well watered (WW) and drought (DRT) treatments. DE genes identified from one-way ANOVA among samples between combination of treatment and developmental stages at Q-value of 1E-6.

Tissue	V12		V14		V18		R1	
	Up-Regulated	Down-Regulated	Up-Regulated	Down-Regulated	Up-Regulated	Down-Regulated	Up-Regulated	Down-Regulated
Leaf	693	999	568	428	749	1203	1684	1499
Ear	875	1608	109	169	1042	503	1219	2624
Tassel	332	362	1961	1077	2	2	1	18

Supplemental Figure S8. Differentially expressed (DE) genes responding to drought stress in vegetative and reproductive tissues, Woodland, CA at different development stages. Genes responding to water treatment were selected at Q-value of 1E-2 from the total pool of DE genes in Supplemental Figure S7.

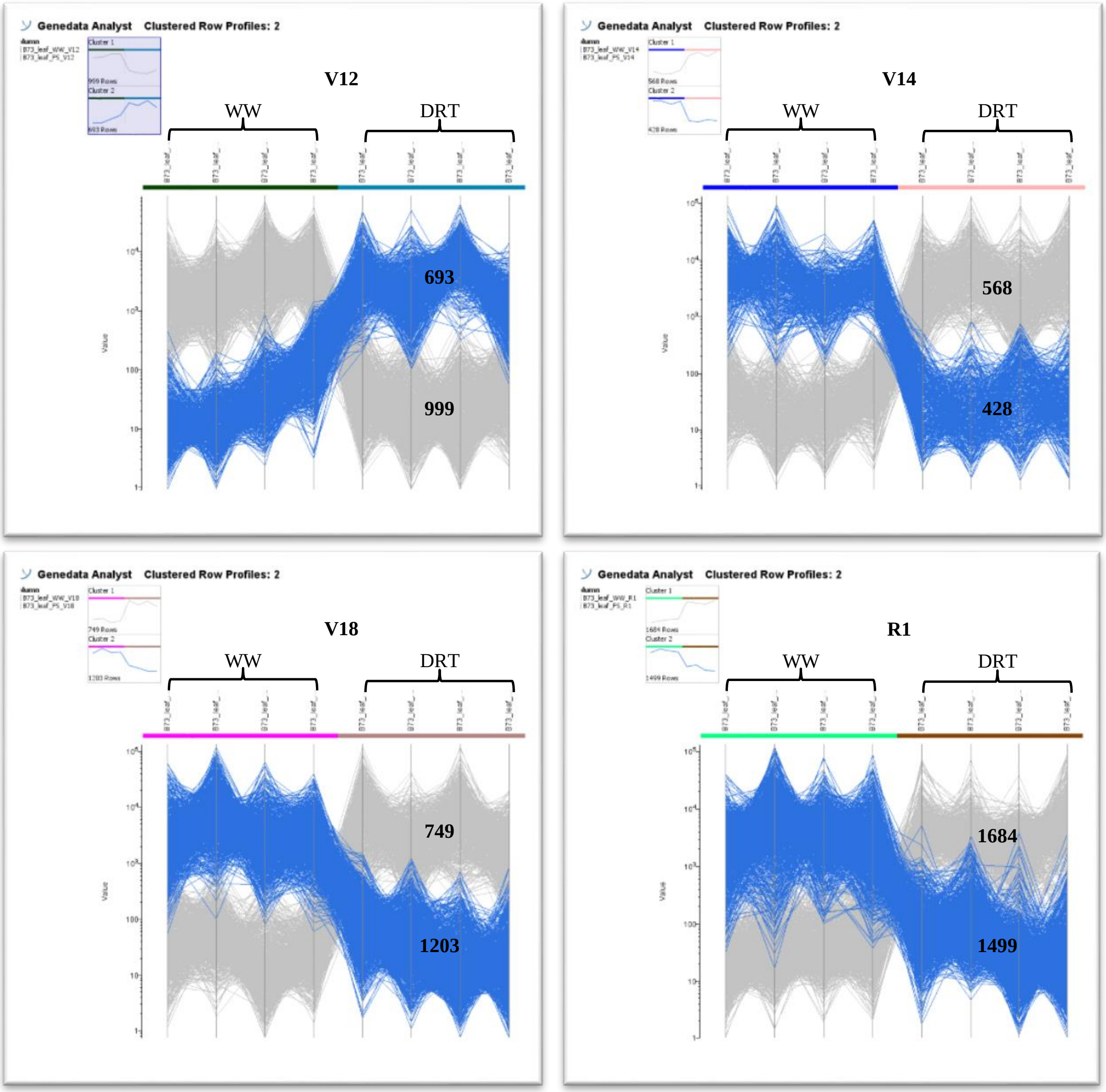

Supplemental Figure S9. K-Means clustering at four development stages (V12, V14, V18, R1) of well-watered (WW) vs drought (DRT) leaf samples. DRT responsive genes were selected at Q-value of 1E-2 from 3454 DE genes (Table V). Up-regulated (top number) and down-regulated (bottom number) genes under DRT by stage.

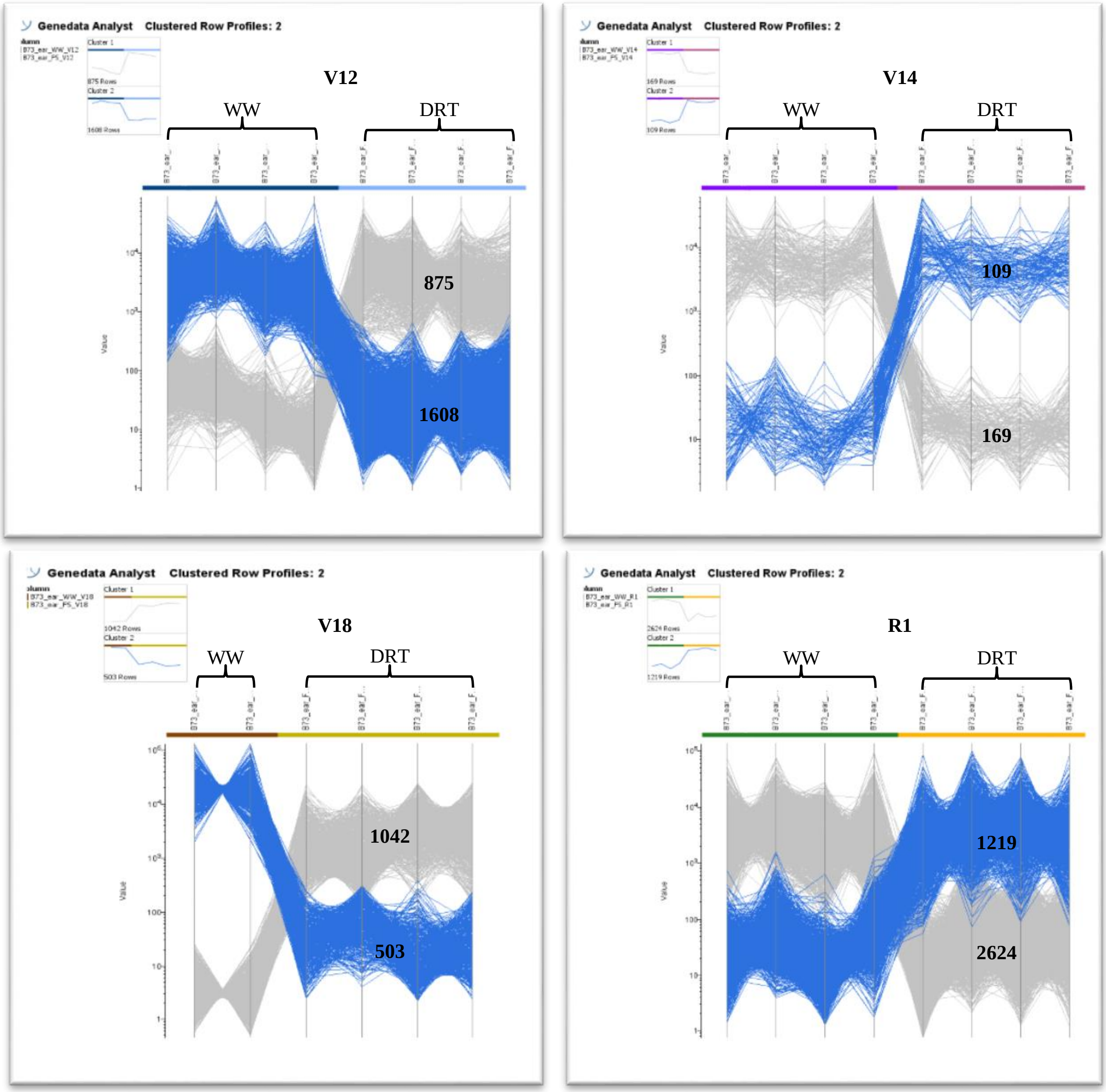

Supplemental Figure S10. K-Means clustering at four development stages (V12, V14, V18, R1) of well-watered (WW) vs drought (DRT) ear samples. DRT responsive genes were selected at Q-value of 1E-2 from 6946 DE genes (Table V) . Up-regulated (top number) and down-regulated (bottom number) genes under DRT by stage.

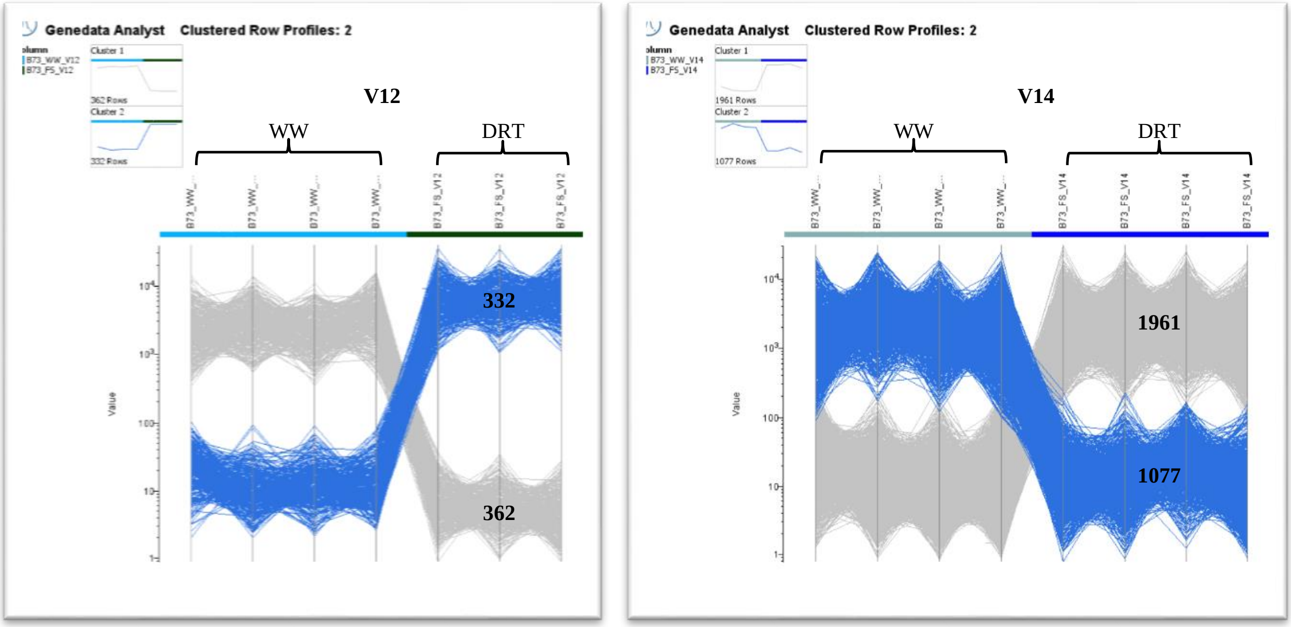

Supplemental Figure S11. K-Means clustering at two development stages (V12, V14) of well-watered (WW) vs drought (DRT) tassel samples. DRT responsive genes were selected at Q-value of 1E-2 from 19850 DE genes (Table V). Up-regulated (top number) and down-regulated (bottom number) genes under DRT by stage.

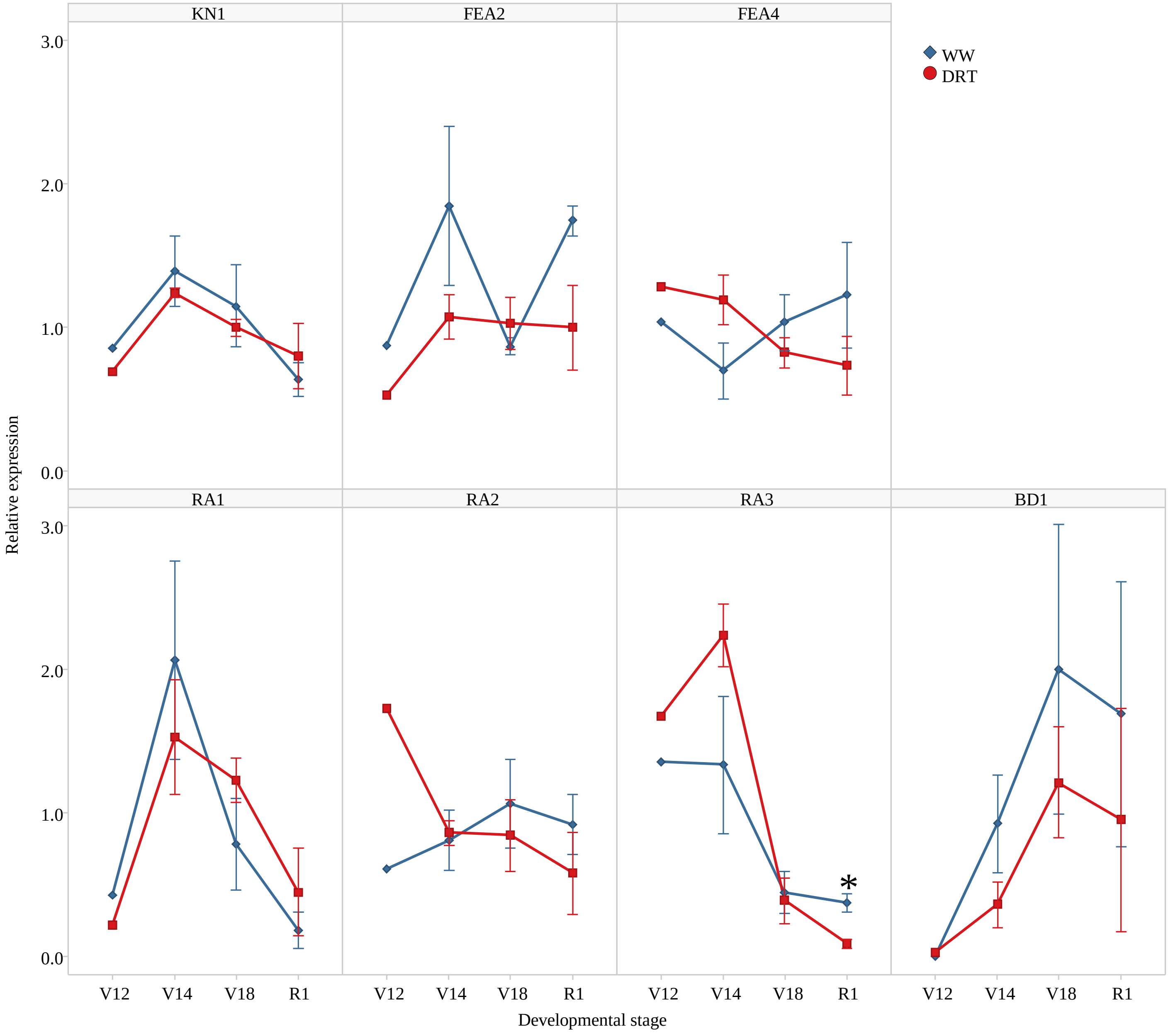

Supplemental Figure S12. Expression of select meristem maintenance genes in developing ears (qRT-PCR). Data points represent means ± SD. Data were measured under well watered (WW) (blue diamond) and drought (DRT) (red square) treatments**. ***Means are statistically different from WW at P < 0.01.

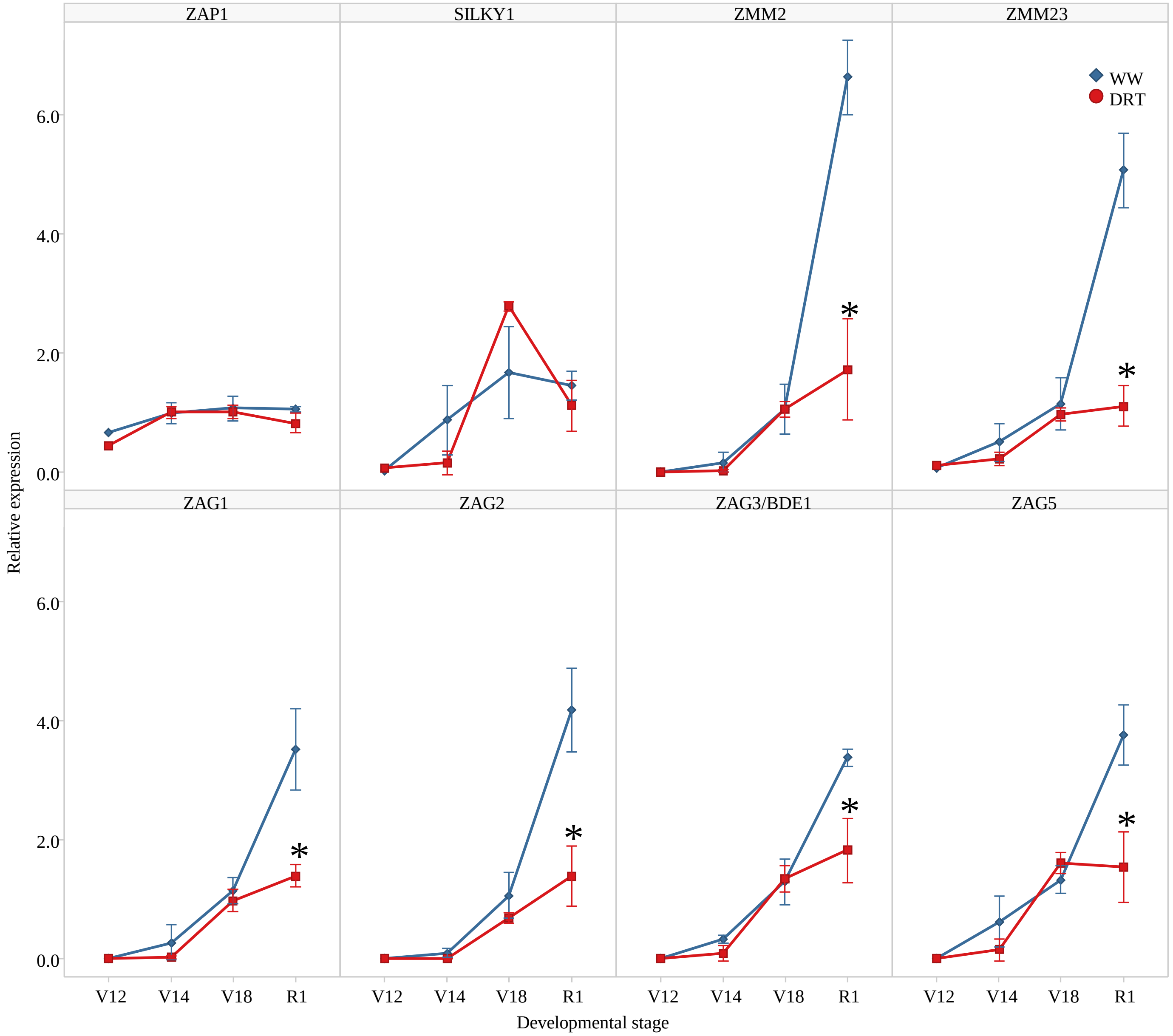

Supplemental Figure S13. Expression of select MADS box genes in developing ears (qRT-PCR). Data points represent means ± SD. Data were measured under well watered (WW) (blue diamond) and drought (DRT) (red square) treatments. *Means are statistically different from WW at P < 0.01.
