# Supplementary material for: Developmental and transcriptional responses of maize to drought stress under field conditions
Source: Plant Direct. 2019 May 7;3(5):e00129. doi: 10.1002/pld3.129 (PMC6589525; doi:10.1002/pld3.129)
Supplement: Supplementary file 5 [file PLD3-3-e00129-s005.pdf]

Reviewer comments:

1. The sampling points describe in Fig. 4, Fig. S1 and Fig. S5 are not consistent. The first sampling point was denoted as V12 in Fig. 4; it was denoted as V10 in Fig. S5; and it was V11-V12 in Fig. S1. Was it V12 under WW, and under DRT it was V11, since the growth rate was decreased by drought stress?

Thank you for pointing out these inconsistencies. As with all field-based experiments, plant growth is not 100% synchronous, therefore the plants were sampled within a narrow growth range - e.g., the first sampling was in the range V10 to V12. In the Methods section, we fully describe the V-stage sampling ranges but for conciseness in the text and figure legends we used only the later V-stage of each range. Thus, the first sampling was from V10-V12 which we designate as V12 in the text. We have made all sampling stages consistent with this designation. The results presented in Figures 4 and S1 refer to the field-based Woodland experiment and the results presented in Fig. S5 pertain to the field pot study, an independent experiment where plants were sampled at different V-stages than the Woodland trial. In addition, within an experiment, the V-stages (as determined by leaf number) under WW and DRT condition were not significantly different (Supplemental Fig. S2A), so the drought treatment had no significant effect on V-stage growth.

2. In M&M, Lines 563-567, the authors described the sampling process. Ears were sampled under WW and DRT at the same time. Were the DRT-ears developmentally delayed? This maybe the reason why the DNA replication and cell division genes were observed repressed by drought, and the genes involved in "regulation of cell cycle" were observed to be upregulated in ear at V18 and R1 stages.

Good point. We did consider this. At the time of sampling, WW and DRT ears were at similar developmental stages as determined by V-growth stage and the number of spikelets produced per ear, even though the DRT ears were smaller. The pot study, which had a more detailed phenotyping assay, confirmed that drought had no significant effect on the total numbers of spikelets produced and a minor reduction in spikelet initiation rate (Fig. 2 and Table III). The small ear size in the DRT is consistent with reduced cell division and/or expansion likely due to reduced expression of DNA replication and cell division genes. We have clarified this better in the Discussion.

3. The authors stated that developmental genes are less affected by drought stress compared with plant growth. However, it is repeatedly described that drought delayed the leaf appearance that "Plant in DRT treatment produced 1-2 fewer leaves that WW plants". "In contrast, plants in the DRT treatment shed pollen three days after the WW plots and no silk exertion was observed. (Page 8, Line 119-122)" Is it not related to plant development? Only the final spikelet number is developmental event? It is very hard to compare the samples harvested on the same day, while one is grown under well-water

conditions, the other is grown under water deficit conditions, since developmentally they are different. It is more comparable to harvest the samples based on the same developmental stage but not on the exactly same date under WW and DRT.

Thank you for pointing this out. In our paper we claim that drought had less of an impact on developmental genes compared to general growth-related genes only in ears. We define “developmental genes” as those primarily involved in the specification and initiation of new organs (leaves, spikelets, etc.) from meristems. Once the organ primordia have formed, they increase in size through the general growth processes of cell division and cell expansion. To clarify this, we have changed “developmental” to “organ initiation” when referring to the general categories of genes affected by drought throughout the paper. This distinction is consistent with our observations that silk exsertion is delayed (a growth effect) but short, unexserted silks are present (an effect of organ initiation) on DRT ears, for example. Moreover, as explained in the response to comment #1, tissue was sampled from plants within a narrow growth range, bulked from 3 plants per replicate, and each treatment had 4 replicates. Thus, the treatment and control plants are within comparable developmental windows at each sampling date that allowed us to identify gene expression differences due to drought.

4. Based on the description, no matter high and low temperature impacted the transcriptome the V12 and V14 samples or not, it should be controlled by such a comparative transcriptomic analysis when comparing with the WW samples which were grown and collected under the same environments. Samples from WW and DRT should only be differed by water stress. The effects of heat can be really revealed by such analyses?

Thank you for the comment. Since our experiment was conducted in the field, we could not control for temperature. Although we were primarily screening for drought-associated gene expression changes, we did observe an unexpected reduction in the number of DE genes during the period of cool temperatures. We agree that to prove this was strictly a temperature response, we need a control group of plants where the temperature remained high. In the revised paper, we simply state the results we observed and make no claims regarding their cause. But we do cite a few articles supporting the idea that the decrease in the number of DE genes might be due to the cooler temperature. And we state that to unambiguously determine the cause of the change in number of DE genes, requires additional studies under controlled environmental conditions.

5. In lines 304-305, "protein folding" and "heat response" were highly enriched in leaves and ears, but not in tassel (Fig. 5A and 5C). Why? It seemed to be contradict with the normal phenomenon that usually male tissue especially pollen is more sensitive to heat

Thank you for pointing this out. The lack of expression of the hallmark stress genes (protein folding and heat response) in tassel tissue suggests that tassels experience less stress than ears and leaves. This observation was unexpected to us as well. However, this finding is consistent with published data. Other studies have shown that tassel growth is less sensitive to stress than ear growth (Herrero P. M. Johnson R.R. Drought Stress and Its Effects on Maize Reproductive Systems (1980) Crop Science: 21, p 105-110). We cited this paper twice in our manuscript. Tassel growth is different from pollen viability too. Other studies have shown that maize pollen viability is more sensitive to heat than to drought, which we have cited. *"Water and heat stress had large effects on ear receptivity and pollen viability, respectively, but pollen viability was unaffected by a water deficit". (Herrero P. M. Johnson R.R. High Temperature Stress and Pollen Viability of Maize (1980) Crop Science: 20, p. 796-800. Schoper, J. B. Lambert R. J. and Vasilas B. L . Maize Pollen Viability and Ear Receptivity under Water and High Temperature Stress 1985, Crop science Vol. 26 No. 5, p. 1029-1033*)

6. In M&M, lines 567, it described that "Visible silks were manually removed from ears at the time of sampling and whole ears were used." The enlarged ASI by drought is probably due to the reduced growth of the silk out of cob under drought stress. Could the authors comment on this?

It is well-established that silk growth under drought slows and results in an increased ASI, a trait that is easily observed by breeders. This is consistent with our observations that at R1, the DRT ears had short silks that had not grown out past the husk leaves, as the WW control silks had. Our study focused on ear tissue since there are several published articles on silk profiling ("*Identification of genes specifically or preferentially expressed in maize silk reveals similarity and diversity in transcript abundance of different dry stigmas BMC Genomics201213:294"; Ovary Apical Abortion under Water Deficit Is Caused by Changes in Sequential Development of Ovaries and in Silk Growth Rate in Maize. Plant Physiol. 2016 Jun;171(2):986-96.)*.

7. During the whole drought treatment and sampling process, the weather temperature also greatly fluctuated, which certainly brought great effects on plant transcriptome, making the study even more complicated.

Thank you for your comment. Please see our response to comment #4. Since both the control and treated plants were experiencing the same temperatures, we can discount any major effect of temperature from our gene expression results. Picking apart the individual and interacting impacts of temperature and drought on gene expression is beyond the scope of our study.
